# The E3 Ligase NEDD4L Prevents Colorectal Cancer Liver Metastasis via Degradation of PRMT5 to Inhibit the AKT/mTOR Signaling Pathway

**DOI:** 10.1002/advs.202504704

**Published:** 2025-04-25

**Authors:** Zhewen Dong, Xiaofei She, Junxian Ma, Qian Chen, Yaqun Gao, Ruiyan Chen, Huanlong Qin, Bing Shen, Hua Gao

**Affiliations:** ^1^ Tongji University Cancer Center and Research Institute of Intestinal Diseases Shanghai Tenth People's Hospital School of Medicine Tongji University Shanghai 200092 P. R. China; ^2^ Shanghai Key Laboratory of Signaling and Disease Research School of Life Sciences and Technology Tongji University Shanghai 200092 P. R. China; ^3^ Department of Urology and Urologic Cancer Institute Shanghai Tenth People's Hospital School of Medicine Tongji University Shanghai 200092 P. R. China

**Keywords:** colorectal cancer liver metastasis, NEDD4L, E3 ubiquitin ligase, PRMT5, AKT/mTOR signaling pathway

## Abstract

Colorectal cancer is the second most common cause of cancer mortality worldwide, and liver metastasis is the major cause of death of patients with colorectal cancer. Dysfunctional E3 ligase activity has recently been shown to be associated with colorectal cancer. However, the key E3 ligases affecting colorectal cancer liver metastasis remain unknown. Therefore, an shRNA library targeting 156 E3 ubiquitin ligases has been used to perform an in vivo loss‐of‐function screen of a human colorectal cancer cell line in a mouse model of liver metastasis. The screen reveals that neural precursor cell expressed developmentally down‐regulated gene 4‐like (NEDD4L) knockdown promotes colorectal cancer liver metastasis. Mechanistic studies reveal that NEDD4L binds to the PPNAY motif in protein arginine methyltransferase 5 (PRMT5) and ubiquitinates PRMT5 to promote its degradation. PRMT5 degradation attenuates the arginine methylation of AKT1 to inhibit the AKT/mTOR signaling pathway. The effect of NEDD4L decreases colorectal cancer cell proliferation to suppress colonization. This study is the first to show that PRMT5 is a substrate of NEDD4L and reveals not only the metastasis‐inhibiting function of NEDD4L but also a novel mechanism by which NEDD4L prevents colorectal cancer liver metastasis. These findings may provide a new preventive strategy for liver metastasis.

## Graphical abstract

Neural precursor cell expressed developmentally down‐regulated gene 4‐like (NEDD4L) prevents colorectal cancer liver metastasis through ubiquitination and degradation of protein arginine methyltransferase 5 (PRMT5). PRMT5 degradation attenuates the methylarginine of AKT1. The effect of NEDD4L inhibits the AKT/mTOR signaling pathway to decrease colorectal cancer cell proliferation, consequently suppressing colonization. This result may provide a potential target for colorectal cancer liver metastasis therapy.

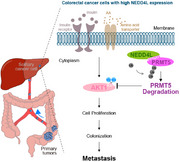

## 1 Introduction

Colorectal cancer is the second most common cause of cancer mortality worldwide and is responsible for ≈1.5–2 million deaths annually.^[^ ^1^ ^]^ More than 50% of patients with colorectal cancer develop liver metastasis, which is the major cause of death of patients with colorectal cancer.^[^ ^2^ ^]^ Ubiquitination is a posttranslational modification.^[^ ^3^ ^]^ E3 ubiquitin ligases, the enzymes that mediate the essential step of ubiquitination, specifically link the ubiquitin protein to substrate proteins. To date, more than 600 E3 ligases have been identified.^[^ ^4^, ^5^ ^]^ Recently, various reports have shown that the dysfunctional activity of E3 ligases is associated with cancer.^[^ ^6^, ^7^, ^8^, ^9^, ^10^ ^]^ However, the key E3 ligases affecting colorectal cancer liver metastasis remain unknown.

Neural precursor cell expressed developmentally down‐regulated gene 4‐like (NEDD4L), a member of the NEDD4 family, is a highly conserved HECT (homologous to E6AP C terminus) E3 ubiquitin ligase.^[^ ^11^ ^]^ NEDD4L can regulate the abundance of proteins that participate in epithelial Na^+^ channel regulation, DNA damage repair, autophagy, and antiviral immunity in a manner dependent mainly on the substrate proteins.^[^ ^12^, ^13^, ^14^, ^15^, ^16^, ^17^, ^18^, ^19^, ^20^, ^21^ ^]^ Although several studies have revealed the downregulation of NEDD4L in colorectal cancer and suggested that NEDD4L is a tumor suppressor,^[^ ^12^, ^14^, ^18^, ^22^ ^]^ the role and mechanism of NEDD4L in colorectal cancer liver metastasis have not been elucidated.

Protein arginine methylation, which is catalyzed by protein arginine methyltransferases (PRMTs), is involved in diverse cellular functions, including cell proliferation and differentiation, RNA splicing, and signal transduction.^[^ ^23^, ^24^ ^]^ Protein arginine methyltransferase 5 (PRMT5), a type II PRMT, catalyzes the formation of monomethylarginine (MMA) and symmetric dimethylarginine (SDMA) in target proteins.^[^ ^25^ ^]^ Accumulating evidence indicates that PRMT5 is upregulated in colorectal cancer and is thus considered an oncogene.^[^ ^26^, ^27^, ^28^ ^]^ Recent studies have shown that PRMT5 is regulated by the ubiquitin–proteasome system. The E3 ligase CHIP ubiquitinates and degrades PRMT5 through K48‐linked ubiquitin chains to inhibit the growth of human prostate cancer cells. K302, K329, K333, K343, K354, K380, and K387 on PRMT5 are mainly involved in this process.^[^ ^29^ ^]^ The E3 ligase UBR7 ubiquitinates and degrades PRMT5 through K48‐linked ubiquitination at the K227 and K240 residues of PRMT5, contributing to gemcitabine resistance in pancreatic cancer.^[^ ^30^ ^]^ Furthermore, E6AP, which also belongs to the HECT family like NEDD4L, ubiquitinates and degrades PRMT5.^[^ ^31^ ^]^ However, in colorectal cancer liver metastasis, the E3 ligase that regulates the ubiquitination and degradation of PRMT5 has not yet been identified.

Here, 794 short hairpin RNAs (shRNAs) targeting 156 E3 ubiquitin ligases were selected to construct an shRNA library (Table S1, Supporting Information), and the library was used to perform an in vivo loss‐of‐function screen of the human colorectal cancer cell line HCT‐15. We focused on metastatic colonization in the liver, the rate‐limiting step of the metastatic cascade.^[^ ^32^ ^]^ Hence, an intrasplenic injection of colorectal cancer cells was performed to establish a mouse model of colorectal cancer liver metastasis for the in vivo functional screen. The screening results revealed that NEDD4L knockdown promoted colorectal cancer liver metastasis. Moreover, we found that the overexpression of NEDD4L prevented colorectal cancer liver metastasis, whereas an E3 ligase activity‐dead mutant of NEDD4L failed to suppress liver metastasis, indicating that the function of NEDD4L depends on its E3 ubiquitin ligase activity. To identify the substrate proteins of NEDD4L, NEDD4L was pulled down, and mass spectrometry was used to analyze the immunoprecipitates. This study revealed that PRMT5 is a substrate protein of NEDD4L. Mechanistically, the NEDD4L‐mediated ubiquitination of PRMT5 promotes its degradation to attenuate the methylarginine of AKT1. This decrease in the methylarginine level of AKT1 leads to the inhibition of the AKT/mTOR signaling pathway and a subsequent decrease in colorectal cancer cell proliferation. Taken together, the results of our study reveal that NEDD4L decreases the proliferation of colorectal cancer cells to suppress colonization, in turn preventing colorectal cancer liver metastasis. This effect relies on the NEDD4L‐induced ubiquitination and degradation of PRMT5 to inhibit the AKT/mTOR signaling pathway.

## 2 Results

### 2.1 Identification of NEDD4L as a Repressor of Colorectal Cancer Liver Metastasis Through an In Vivo Functional Screen

**Figure 1:**
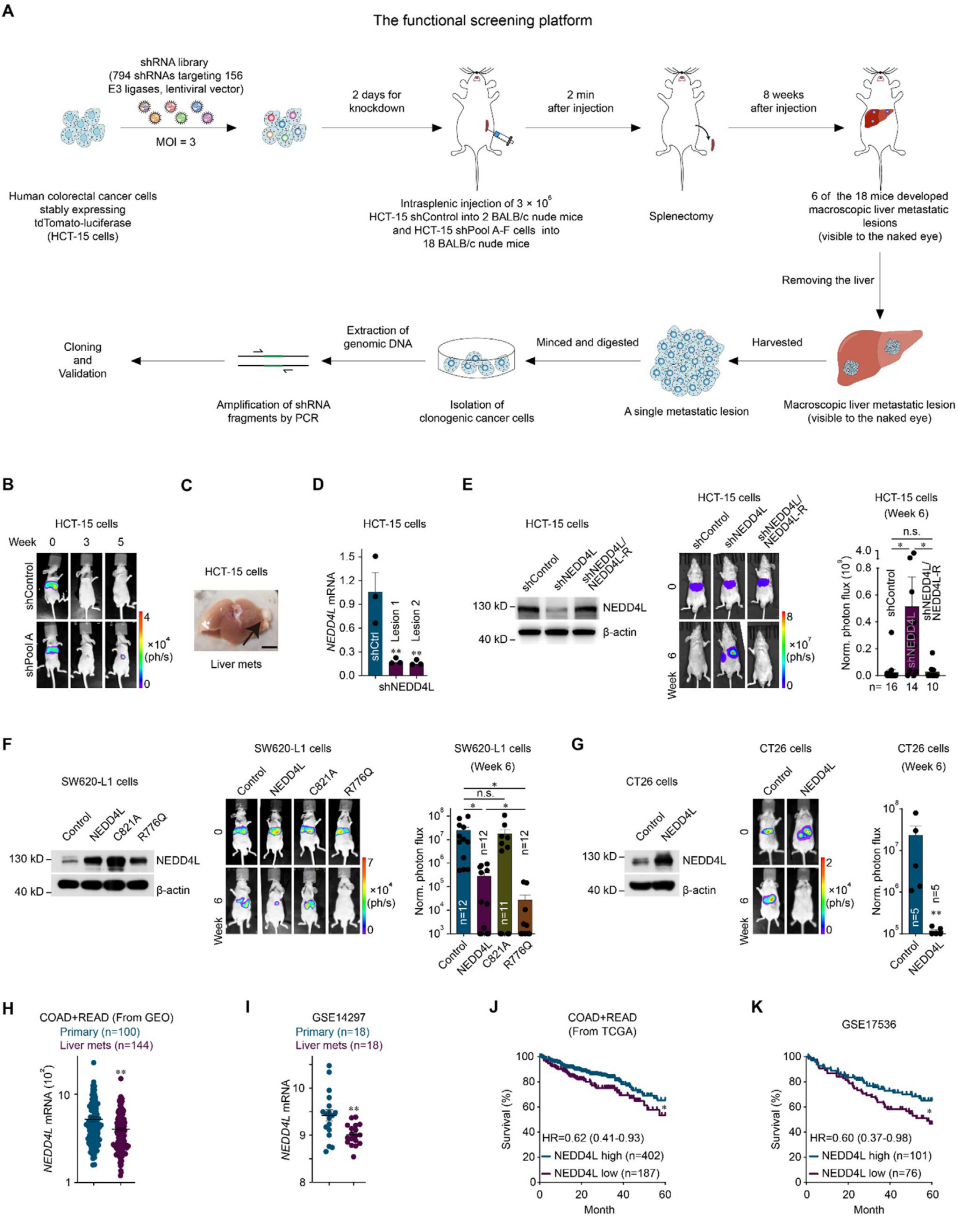
Identification of NEDD4L as a repressor of colorectal cancer liver metastasis through an in vivo functional screen. A) Schematic of the loss‐of‐function screening strategy based on an shRNA library used to identify genes that repress colorectal cancer liver metastasis. B) Representative bioluminescence images of BALB/c nude mice implanted with HCT‐15 human colorectal cancer cells transduced with shControl or the shRNA library targeting E3 ubiquitin ligases (3 × 10^6^ cells; shPool A containing shNEDD4L; shControl: 2 mice and shPool A: 3 mice). C) Representative screening results for liver metastatic lesions in BALB/c nude mice implanted with HCT‐15 cells transduced with shPool A (3 × 10^6^ cells; shPool A containing shNEDD4L). The arrow indicates liver lesions (macroscopically visible). Scale bar, 1 cm. D) NEDD4L mRNA level in HCT‐15 cells recovered from two liver metastatic lesions containing shNEDD4L that were formed by HCT‐15 cells transduced with shPool A. E) Representative western blots showing NEDD4L expression in control HCT‐15 cells (shControl), NEDD4L‐knockdown HCT‐15 cells (shNEDD4L) and HCT‐15 cells with knockdown of NEDD4L and restoration with wild‐type NEDD4L resistant to shNEDD4L targeting (shNEDD4L/NEDD4L‐R) (left). Bioluminescence imaging results (middle) and quantification of liver metastases (right) in BALB/c nude mice implanted with shControl, shNEDD4L or shNEDD4L/NEDD4L‐R HCT‐15 cells (3 × 10^6^ cells) via intrasplenic injection. The *n*‐ values denote the number of mice per group. F) Representative western blots showing NEDD4L and mutant NEDD4L expression in control SW620‐L1 cells (Control), and SW620‐L1 cells overexpressing wild‐type NEDD4L (NEDD4L), an E3 ligase activity‐dead mutant of NEDD4L (C821A) or a constitutively active mutant of NEDD4L (R776Q) (left). Bioluminescence imaging results (middle) and quantification of liver metastases (right) in BALB/c nude mice implanted with Control, NEDD4L, C821A or R776Q SW620‐L1 cells (1 × 10^6^ cells) via intrasplenic injection. The *n*‐ values denote the number of mice per group. G) Representative western blots showing NEDD4L expression in Control CT26 cells or NEDD4L CT26 cells (left). Bioluminescence imaging results (middle) and quantification of liver metastases (right) in BALB/c mice implanted with Control or NEDD4L CT26 cells (3 × 10^5^ cells) via intrasplenic injection. The *n*‐ values denote the number of mice per group. H) NEDD4L mRNA expression in the primary tumors and unpaired liver metastatic lesions of patients with colorectal cancer in the combined dataset from the GLP570 platform (Affymetrix Human Genome U133 Plus 2.0 Array). The combined dataset consists of GSE10961, GSE18462, GSE28702, GSE40367 and GSE41568. The *n*‐ values denote the number of patients per group. I) NEDD4L mRNA expression in the primary tumors and paired liver metastatic lesions of patients with colorectal cancer in the GSE14297 dataset. The *n*‐ values denote the number of patients per group. J,K) Kaplan‒Meier curves of 5‐year survival for patients with colorectal cancer represented in TCGA datasets (COAD and READ, n = 589, J) and the GSE17536 dataset (n = 177, K) stratified by the NEDD4L mRNA expression level. HR, hazard ratio. Three independent experiments were performed (D, protein expression detection in E‐G). The data are presented as the mean ± s.e.m. values. *P*‐ values were determined by unpaired one‐way ANOVA with uncorrected Fisher's LSD test (D‐F), unpaired two‐tailed Student's t‐test with Welch's correction (G), the Wilcoxon rank‐sum test (H, I), or the log‐rank test (J, K). * *P* < 0.05; ** *P* < 0.01; n.s., not significant.

A total of 794 shRNAs targeting 156 cancer‐related E3 ligases were selected to construct an shRNA library (Table S1, Supporting Information), and the shRNA library was used to perform an in vivo loss‐of‐function screen of the human colorectal cancer cell line HCT‐15 to identify the key E3 ubiquitin ligases that prevent colorectal cancer liver metastasis (**Figure** **1**A). We focused on metastatic colonization in the liver, the rate‐limiting step of the metastatic cascade.^[^ ^32^ ^]^ Hence, an intrasplenic injection of colorectal cancer cells was performed to establish a mouse model of colorectal cancer liver metastasis for the in vivo functional screen (Figure 1A).

Five candidate genes (NEDD4L, BMI, SMURF1, AREL1 and FBXW2) were identified from the screen. shNEDD4L (ID: TRCN0000000905), contained in shPool A, was identified in two independent liver metastatic lesions derived from two different mice (Figure 1B,C; Figure S1A, Supporting Information). The indicence of liver metastasis was 66.67% (2 of 3 mice) for NEDD4L‐knockdown HCT‐15 cells, whereas it was 33.33% (1 of 3 mice) for BMI‐, SMURF1‐, AREL1‐, and FBXW2‐knockdown HCT‐15 cells (Figure S1A, Supporting Information). Therefore, we considered shNEDD4L the top‐ranked shRNA. Next, NEDD4L knockdown in HCT‐15 cells recovered from the 2 liver metastatic lesions was confirmed at the transcriptional level (Figure 1D). Notably, among the five NEDD4L shRNAs in the library, one shRNA (shNEDD4L, ID: TRCN0000000905) was identified from the screen and had the highest knockdown efficiency (Figure S1B, Supporting Information).

Consistent with the screening results, in the validation experiment, knockdown of NEDD4L with shNEDD4L (TRCN0000000905) promoted the liver metastasis of HCT‐15 cells (Figure 1E). To prevent any off‐target effect of shNEDD4L, NEDD4L‐R, which is resistant to shNEDD4L (TRCN0000000905) targeting, was used to restore NEDD4L expression in NEDD4L‐knockdown colorectal cancer cells, since we did not find in our system that the knockdown efficiency of any published shRNA (different from the 5 shRNAs in the library) was similar to that of shNEDD4L (TRCN0000000905). In the functional rescue experiment, the overexpression of NEDD4L‐R in NEDD4L‐knockdown colorectal cancer cells prevented liver metastasis, suggesting that restoring NEDD4L expression reversed the effect of NEDD4L knockdown on colorectal cancer liver metastasis (Figure 1E). Moreover, in SW620‐L1 and CT26 cells, which are highly metastatic colorectal cancer cell lines derived from humans and mice, respectively, the overexpression of wild‐type NEDD4L prevented the liver metastasis of cancer cells (Figure 1F,G; Figure S1C, Supporting Information).

An E3 ligase activity‐dead mutant of NEDD4L (NEDD4L C821A) and a constitutively active mutant of NEDD4L (NEDD4L R776Q) ^[^ ^33^ ^]^ were generated and overexpressed in SW620‐L1 cells to determine whether the E3 ligase activity of NEDD4L plays a critical role in its ability to inhibit metastasis (Figure 1F; Figure S1D, Supporting Information). NEDD4L C821A overexpression failed to prevent colorectal cancer liver metastasis, and NEDD4L R776Q overexpression prevented colorectal cancer liver metastasis more potently than did NEDD4L wild‐type overexpression (Figure 1F), suggesting that the E3 ligase activity of NEDD4L is important for its ability to prevent metastasis.

Additionally, in our analysis of clinical samples, although NEDD4L expression was high in primary tumors, it was lower in liver metastatic lesions (Figure 1H,I). Moreover, low expression of NEDD4L in primary tumors was correlated with poor 5‐year overall survival outcomes in patients with colorectal cancer (Figure 1J,K).

### 2.2 NEDD4L Decreasing Cell Proliferation Suppresses Colonization to Prevent Colorectal Cancer Liver Metastasis Through the Inhibition of the AKT/mTOR Signaling Pathway

**Figure 2:**
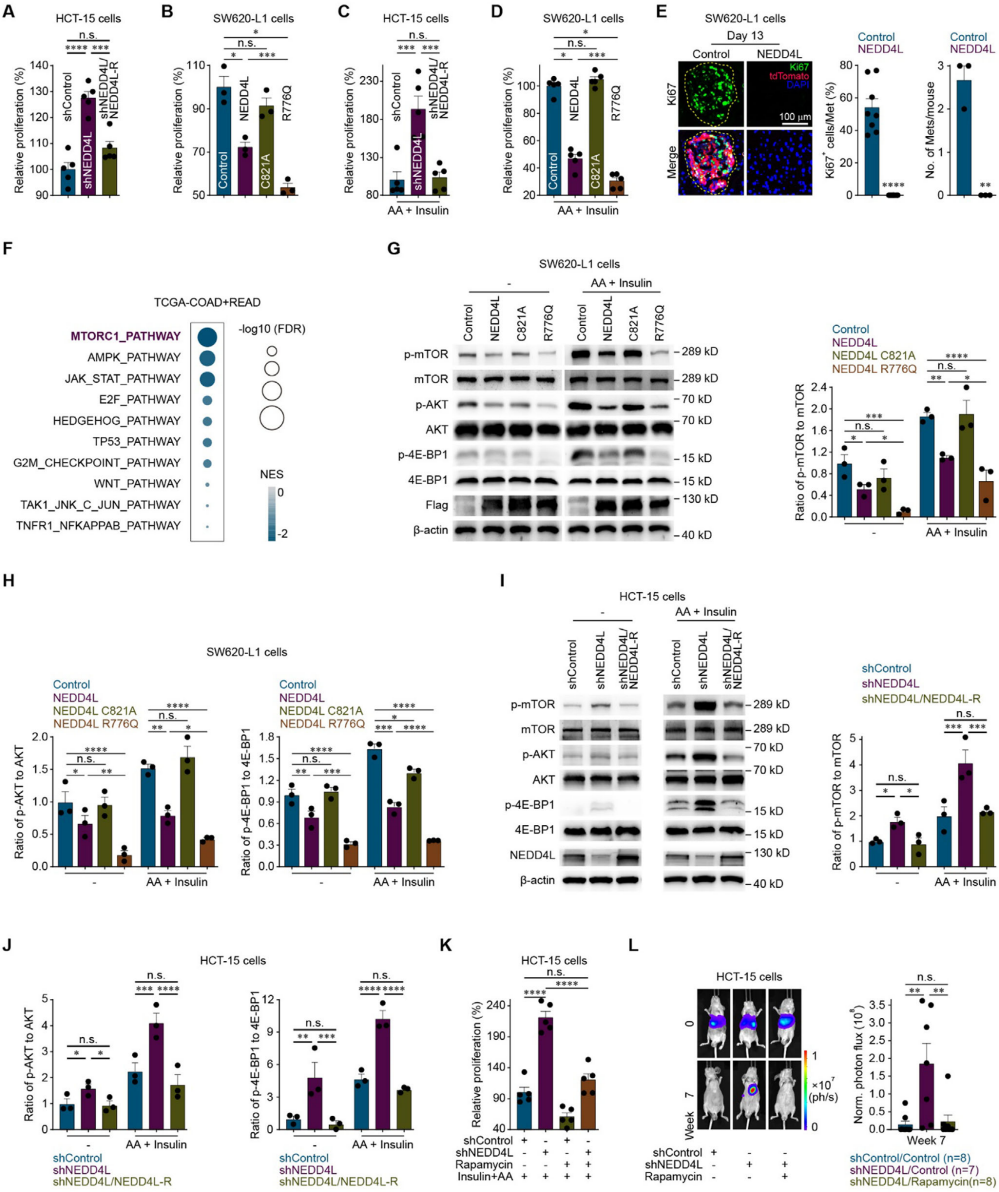
NEDD4L decreasing cell proliferation suppresses colonization to prevent colorectal cancer liver metastasis through the inhibition of the AKT/mTOR signaling pathway. A) In vitro proliferation assay of shControl, shNEDD4L or shNEDD4L/NEDD4L‐R HCT‐15 cells (1000 cells) cultured for 72 h. B) In vitro proliferation assay of Control, NEDD4L, NEDD4L C821A or NEDD4L R776Q SW620‐L1 cells (3000 cells) induced with 2 µg mL^−1^ doxycycline and cultured for 72 h. Three independent experiments were performed. C) In vitro proliferation assay of shControl, shNEDD4L or shNEDD4L/NEDD4L‐R HCT‐15 cells (1000 cells) cultured with 200 μм amino acids (AA) and 800 nм insulin for 24 h. D) In vitro proliferation assay of control Control, NEDD4L, NEDD4L C821A or NEDD4L R776Q SW620‐L1 cells (3000 cells) induced with 2 µg mL^−1^ doxycycline and cultured with 200 μм AA and 800 nм insulin for 24 h. E) Representative images (left), quantification of Ki67 (green)‐positive SW620‐L1 cells (tdTomato) in the liver metastatic lesions of SW620‐L1 cell‐implanted BALB/c nude mice (middle), and quantification of liver metastatic lesions (right) on day 13 after the intrasplenic injection of Control or NEDD4L SW620‐L1 cells (1 × 10^6^ cells). Eight liver metastatic lesions in 3 mice in the control group, and 0 liver metastatic lesions in 3 mice in the NEDD4L group. Scale bar, 100 µm. Yellow dotted lines (ROIs) or Met, liver metastatic lesions; ROI, region of interest. F) GSEA revealed an association between NEDD4L expression and the enrichment of genes in proliferation‐related signaling pathways in clinical colorectal cancer samples from TCGA datasets (COAD and READ, n = 589). GSEA, gene set enrichment analysis. NES, normalized enrichment score. FDR, false discovery rate. G,H) Representative western blots (G, left) and quantification of the p‐mTOR, mTOR, p‐AKT, AKT, p‐4E‐BP1 and 4E‐BP1 levels (G, right and H) in Control, NEDD4L, NEDD4L C821A or NEDD4L R776Q SW620‐L1 cells induced with 2 µg mL^−1^ doxycycline for 24 h and incubated with or without 200 μм AA for 15 min and 800 nм insulin for 10 min. Three independent experiments were performed. I,J) Representative western blots (I, left) and quantification of the p‐mTOR, mTOR, p‐AKT, AKT, p‐4E‐BP1 and 4E‐BP1 levels (I, right and J) in shControl, shNEDD4L or shNEDD4L/NEDD4L‐R HCT‐15 cells incubated with or without 200 μм AA for 15 min and 800 nм insulin for 10 min. Three independent experiments were performed. K) In vitro proliferation assay of shControl or shNEDD4L HCT‐15 cells (1000 cells) cultured with or without 100 nм rapamycin in combination with 200 μм AA and 800 nм insulin for 24 h. L) Bioluminescence imaging results (left) and quantification of liver metastases (right) in BALB/c nude mice implanted with shControl or shNEDD4L HCT‐15 cells (3 × 10^6^ cells) via intrasplenic injection. Rapamycin (2 mg kg^−1^) was administered intraperitoneally every two days from the day of cancer cell injection (day 0) to the experimental endpoint. The *n*‐ values denote the number of mice per group. Five independent experiments were performed except as specifically pointed out (A, C, D, and K). The data are presented as the mean ± s.e.m. values. *P*‐ values were determined by unpaired one‐way ANOVA with uncorrected Fisher's LSD test (A‐D, K, and L), unpaired two‐tailed Student's t‐test with Welch's correction (E), or unpaired two‐way ANOVA with uncorrected Fisher's LSD test (G‐J). * *P* < 0.05; ** *P* < 0.01; *** *P* < 0.001; **** *P* < 0.0001; n.s., not significant.

The function of NEDD4L related to metastatic colonization was explored since colorectal cancer cells were injected directly into the bloodstream. The in vitro results revealed that the proliferation of colorectal cancer cells was decreased by NEDD4L, although the epithelial‒mesenchymal transition (EMT), invasion and stemness of the colorectal cancer cells were not affected (**Figure** **2**A,B; Figure S2A–E, Supporting Information). The proliferation of colorectal cancer cells stimulated with amino acids (AA) and insulin was subsequently examined since the liver is the main organ that stores AA and insulin.^[^ ^34^, ^35^ ^]^ The proliferation of colorectal cancer cells stimulated with AA and insulin was also decreased by NEDD4L (Figure 2C,D). However, the overexpression of NEDD4L C821A (an E3 ligase activity‐dead mutant) failed to decrease the proliferation of colorectal cancer cells, and the overexpression of NEDD4L R776Q (a constitutively active mutant) increased the suppressive effect of NEDD4L on the proliferation of colorectal cancer cells (Figure 2B,D). Moreover, the in vivo results revealed that NEDD4L overexpression decreased the proliferation of liver‐metastatic colorectal cancer cells (Figure 2E) and that NEDD4L knockdown increased the proliferation of liver‐metastatic colorectal cancer cells (Figure S2F, Supporting Information).

A gene set enrichment analysis (GSEA) of data from patients with colorectal cancer revealed that the activity of the mTOR signaling pathway was negatively correlated with the expression of NEDD4L (Figure 2F). Since AA and insulin are the main activators of the AKT/mTOR signaling pathway,^[^ ^36^, ^37^ ^]^ the effects of NEDD4L on the AKT/mTOR signaling pathway in the context of AA and insulin stimulation were subsequently examined. NEDD4L inhibited the AKT/mTOR signaling pathway regardless of stimulation with AA and/or insulin (Figure 2G,H; Figures S2G,H and S3A–F, Supporting Information), whereas NEDD4L did not affect the AKT/mTOR signaling pathway under stimulation with glucose (Figure S3G,H, Supporting Information). In contrast, NEDD4L knockdown upregulated the AKT/mTOR signaling pathway under both basal and activating conditions (Figure 2I,J; Figure S4A–F, Supporting Information). The functional rescue experiment revealed that overexpression of NEDD4L‐R in NEDD4L‐knockdown HCT‐15 cells inhibited the AKT/mTOR signaling pathway, suggesting that restoring NEDD4L expression reversed the effect of NEDD4L knockdown on the AKT/mTOR signaling pathway (Figure 2I,J). In addition, overexpression of the E3 ligase activity‐dead NEDD4L C821A mutant failed to inhibit the AKT/mTOR signaling pathway, and overexpression of the constitutively active NEDD4L R776Q mutant inhibited the AKT/mTOR signaling pathway more potently than did the overexpression of wild‐type NEDD4L (Figure 2G,H). These results suggested that NEDD4L inhibited the AKT/mTOR signaling pathway and that this effect was dependent on its E3 ligase activity.

Furthermore, in vitro experiments revealed that the effect of NEDD4L knockdown on cancer cell proliferation was reversed by treatment with rapamycin, a selective mTOR inhibitor that suppresses the AKT/mTOR signaling pathway (Figure 2K; Figure S4G, Supporting Information). Consistent with the results of the in vitro experiments, the effect of NEDD4L knockdown on the liver metastasis of HCT‐15 cells was reversed by rapamycin treatment (Figure 2L). These results suggested that NEDD4L decreasing cancer cell proliferation suppressed colonization through the inhibition of the AKT/mTOR signaling pathway to prevent the liver metastasis of colorectal cancer cells.

### 2.3 NEDD4L Ubiquitinates PRMT5 to Promote PRMT5 Degradation

**Figure 3:**
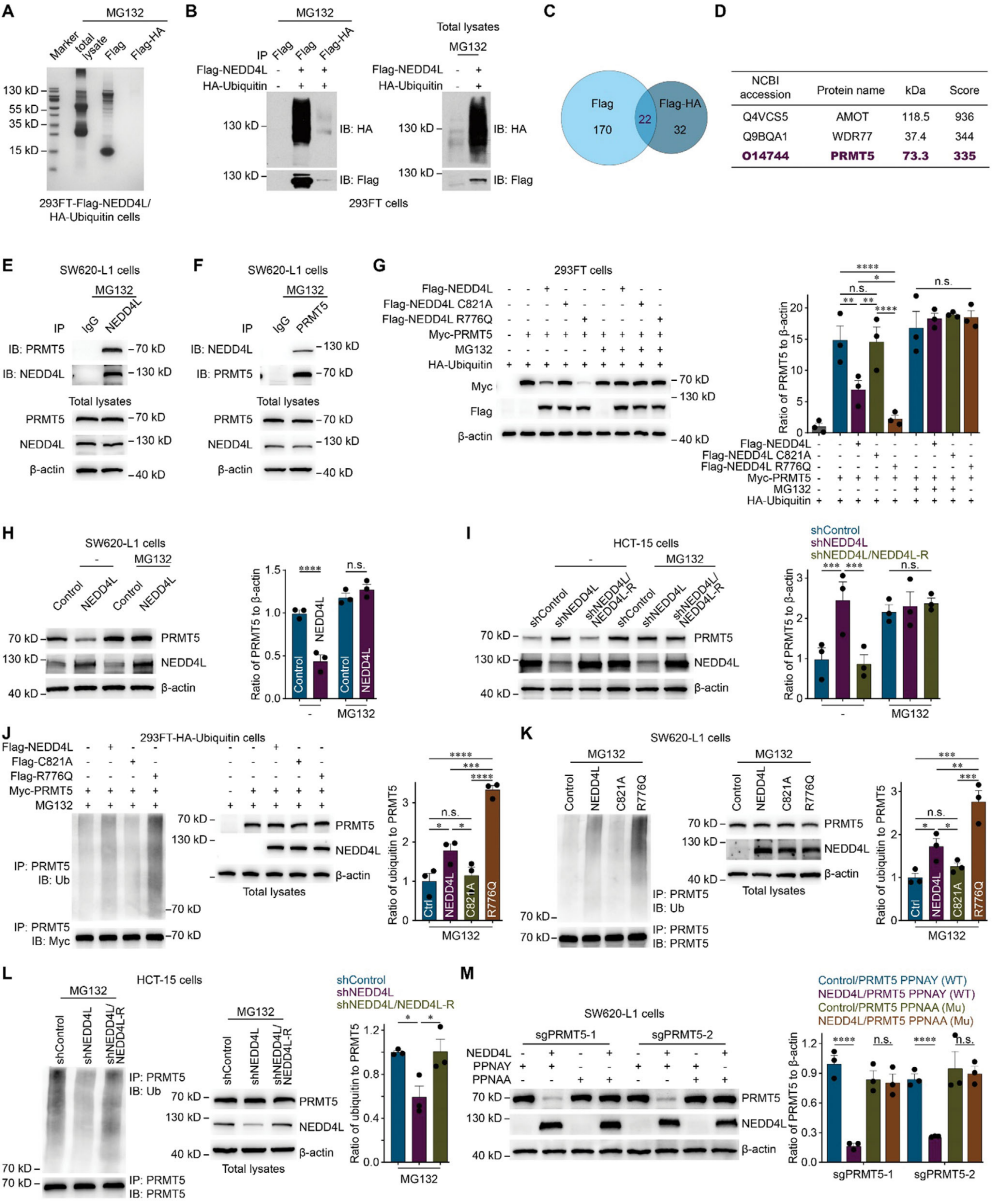
NEDD4L ubiquitinates PRMT5 to promote its degradation. A) EZBlue staining of gels containing the total lysate and the immunoprecipitates of 293FT cells overexpressing Flag‐NEDD4L and HA‐ubiquitin (Flag‐NEDD4L/HA‐ubiquitin) that were incubated with 20 μм MG132 for 12 h. The immunoprecipitates were pulled down with Flag or Flag and HA agarose. B) Representative western blots of proteins immunoprecipitated from Control/Control or Flag‐NEDD4L/HA‐ubiquitin 293FT cells that were incubated with 20 μм MG132 for 12 h and pulled down with Flag or Flag and HA agarose. C) Venn diagram showing the overlap among the candidate proteins identified by mass spectrometry analysis of immunoprecipitates from Flag‐NEDD4L/HA‐ubiquitin 293FT cells incubated with 20 μм MG132 for 12 h and pulled down with Flag or Flag and HA agarose. D) List of the top 3 candidate substrate proteins of NEDD4L identified via mass spectrometry analysis. E,F) Co‐IPs of endogenous NEDD4L and PRMT5 pulled down by NEDD4L (E) or PRMT5 (F) in SW620‐L1 cells. The cancer cells were incubated with 20 μм MG132 for 12 h. G) Representative western blots (left) and quantification of Myc‐PRMT5 expression (right) in Control/Control, Control/Myc‐PRMT5, Flag‐NEDD4L/Myc‐PRMT5, Flag‐NEDD4L C821A/Myc‐PRMT5 or Flag‐NEDD4L R776Q/Myc‐PRMT5 293FT cells with overexpressing HA‐ubiquitin incubated with or without 20 μм MG132 for 12 h. H) Representative western blots (left) and quantification of PRMT5 expression (right) in Control or NEDD4L SW620‐L1 cells induced with 2 µg mL^−1^ doxycycline for 24 h and incubated with or without 20 μм MG132 for 12 h. I) Representative western blots (left) and quantification of PRMT5 expression (right) in shControl, shNEDD4L or shNEDD4L/NEDD4L‐R HCT‐15 cells incubated with or without 20 μм MG132 for 12 h. J) Representative western blots (left) and quantification of PRMT5 ubiquitination and PRMT5 expression (right) in Control/Control, Control/Myc‐PRMT5, Flag‐NEDD4L/Myc‐PRMT5, Flag‐NEDD4L C821A/Myc‐PRMT5 or Flag‐NEDD4L R776Q/Myc‐PRMT5 293FT cells with HA‐ubiquitin overexpression incubated with or without 20 μм MG132 for 12 h. K) Representative western blots (left) and quantification of PRMT5 ubiquitination and PRMT5 expression (right) in Control, NEDD4L, NEDD4L C821A or NEDD4L R776Q SW620‐L1 cells induced with 2 µg mL^−1^ doxycycline for 24 h and incubated with 20 μм MG132 for 12 h. L) Representative western blots (left) and quantification of PRMT5 ubiquitination (right) in shControl, shNEDD4L or shNEDD4L/NEDD4L‐R HCT‐15 cells incubated with or without 20 μм MG132 for 12 h. M) Representative western blots (left) and quantification of exogenous Flag‐PRMT5 and its mutants (right) in Control SW620‐L1 cells or NEDD4L SW620‐L1 cells with the knockout of endogenous PRMT5 (sgPRMT5‐1 or sgPRMT5‐2) and overexpression of wild‐type PRMT5 (PPNAY) or the mutant NEDD4L binding motif (PPNAA). To induce the overexpression of NEDD4L in these cells, the cells were induced with 2 µg mL^−1^ doxycycline for 24 h. Three independent experiments were performed (A, B, and E‐M). The data are presented as the mean ± s.e.m. values. *P*‐ values were determined by unpaired two‐way ANOVA with uncorrected Fisher's LSD test (G‐I and M), or unpaired one‐way ANOVA with uncorrected Fisher's LSD test (J‐L). * *P* < 0.05; ** *P* < 0.01; *** *P* < 0.001; **** *P* < 0.0001; n.s., not significant.

As described above, NEDD4L inhibited the AKT/mTOR signaling pathway in a manner dependent on its E3 ligase activity. Next, we examined whether proteins in the AKT/mTOR signaling pathway are direct substrates of NEDD4L. The results showed that NEDD4L had no effect on the protein levels of AKT, TSC2, or Rheb (Figure S5A,B, Supporting Information). To identify the direct substrate proteins, Flag‐NEDD4L and HA‐ubiquitin were pulled down (**Figure** **3**A,B), and the immunoprecipitates were analyzed by mass spectrometry (MS). The PRMT5–WDR77 complex was identified as 2 top candidate proteins (Figure 3C,D; Table S2, Supporting Information). PRMT5 has been reported to activate AKT,^[^ ^27^, ^38^ ^]^ and the presence of WDR77 as a cofactor can increase the affinity of PRMT5 for its target proteins.^[^ ^39^ ^]^ A validation experiment revealed that NEDD4L interacted with PRMT5 (Figure 3E,F; Figure S5C,D, Supporting Information) and that NEDD4L ubiquitinated PRMT5 to promote its degradation (Figure 3G–L; Figure S5E,F, Supporting Information), which depended on the E3 ligase activity of NEDD4L (Figure 3G,J,K). However, NEDD4L did not affect the stability of WDR77 (Figure S5E, Supporting Information), indicating that WDR77 is not a direct substrate protein of NEDD4L.

NEDD4L binds to PPxY motifs in its substrate proteins via its WW domain.^[^ ^40^ ^]^ Motif mutants of PRMT5 (PPNAA and PANAY) were used to confirm the function of the PPNAY motif in PRMT5 (Figure S5G,H, Supporting Information). In cells in which endogenous endogenous PRMT5 was knocked out, NEDD4L failed to promote the degradation of either of these PRMT5 mutants (Figure 3M; Figure S5G,H, Supporting Information). These results suggested that NEDD4L ubiquitinated PRMT5 to promote its degradation and that this ability was dependent on the PPNAY motif in PRMT5.

### 2.4 PRMT5 Methylates AKT1 at R391 to Activate the AKT/mTOR Signaling Pathway in Colorectal Cancer Cells

**Figure 4:**
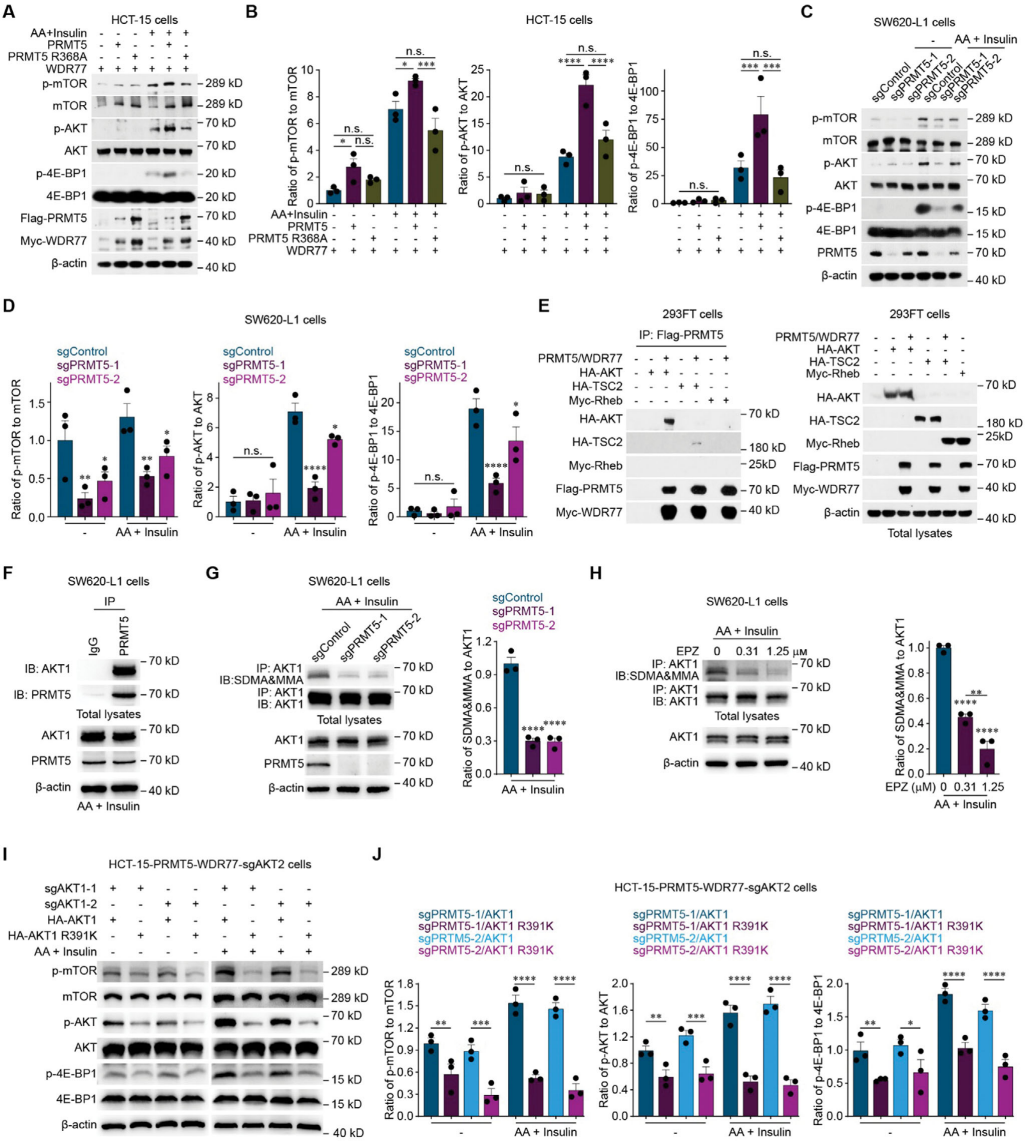
PRMT5 methylates AKT1 at R391 to activate the AKT/mTOR signaling pathway in colorectal cancer cells. A,B) Representative western blots (A) and quantification of the p‐mTOR, mTOR, p‐AKT, AKT, p‐4E‐BP1 and 4E‐BP1 levels (B) in Control HCT‐15 cells or HCT‐15 cells with overexpressing WDR77 in combination with wild‐type PRMT5 (PRMT5/WDR77) or a methyltransferase‐inactive mutant of PRMT5 (PRMT5 R368A/WDR77) incubated with or without 200 μм AA for 15 min and 800 nм insulin for 10 min. C,D) Representative western blots (C) and quantification of the p‐mTOR, mTOR, p‐AKT, AKT, p‐4E‐BP1 and 4E‐BP1 levels (D) in sgControl, sgPRMT5‐1 or sgPRMT5‐2 SW620‐L1 cells incubated with or without 200 μм AA for 15 min and 800 nм insulin for 10 min. E) Representative western blots of Flag‐PRMT5 immunoprecipitates and total lysates from control 293FT cells or 293FT cells overexpressing HA‐AKT, HA‐TSC2, or Myc‐Rheb with or without Flag‐PRMT5 and Myc‐WDR77 overexpression. F) Representative western blots showing PRMT5 immunoprecipitates and total lysates of SW620‐L1 cells incubated with 200 μм AA for 15 min and 800 nм insulin for 10 min. G) Representative western blots (left) and quantification of AKT1 methylarginine and AKT1 levels (right) in sgControl, sgPRMT5‐1, or sgPRMT5‐2 SW620‐L1 cells incubated with 200 μм AA for 15 min and 800 nм insulin for 10 min. H) Representative western blots (left) and quantification of AKT1 methylarginine and AKT1 levels (right) in SW620‐L1 cells treated with EPZ015666 (0, 0.31 or 1.25 μм) for 48 h and incubated with 200 μм AA for 15 min and 800 nм insulin for 10 min. I,J) Representative western blots (I) and quantification of the p‐mTOR, mTOR, p‐AKT, AKT, p‐4E‐BP1 and 4E‐BP1 levels (J) in HCT‐15 cells with PRMT5 overexpression in combination with the double knockout of endogenous AKT1 and AKT2 and overexpression of wild‐type AKT1 (PRMT5/sgAKT1‐1/sgAKT2/AKT1 or PRMT5/sgAKT1‐2/sgAKT2/AKT1) or the AKT1 R391K mutant that cannot be methylated by PRMT5 (PRMT5/sgAKT1‐1/sgAKT2/AKT1‐R391K or PRMT5/sgAKT1‐2/sgAKT2/AKT1‐R391K) incubated with 200 μм AA for 15 min and 800 nм insulin for 10 min. Three independent western blot analyses were performed (A‐J). The data are presented as the mean ± s.e.m. values. *P*‐ values were determined by unpaired two‐way ANOVA with uncorrected Fisher's LSD test (B, D and J), or unpaired one‐way ANOVA with uncorrected Fisher's LSD test (G, H). * *P* < 0.05; **, *P* < 0.01; *** *P* < 0.001; **** *P* < 0.0001; n.s., not significant.

Recent studies have indicated that PRMT5 methylates and activates AKT and consequently increases cancer cell proliferation.^[^ ^27^, ^38^ ^]^ Consistent with published studies, the overexpression of PRMT5 activated the AKT/mTOR signaling pathway (**Figure** **4**A,B; Figure S6A–F, Supporting Information), whereas the overexpression of PRMT5 R368A (a methyltransferase‐inactive mutant of PRMT5) failed to activate the AKT/mTOR signaling pathway (Figure 4A,B). Both the knockout and knockdown of PRMT5, as well as the inhibition of PRMT5 by EPZ015666, a selective PRMT5 inhibitor that targets the substrate binding pocket of PRMT5,^[^ ^41^ ^]^ inhibited the AKT/mTOR signaling pathway (Figure 4C,D; Figure S6G‐J, Supporting Information). Methylation of arginine 391 (R391) in AKT1 has been reported to play a crucial role in AKT activation.^[^ ^27^ ^]^ Our experiments revealed that PRMT5 interacted with AKT1 (Figure 4E,F) and methylated the arginine residue in AKT1 to activate AKT (Figure 4G,H; Figure S7A, Supporting Information), whereas other proteins in the AKT/mTOR signaling pathway did not interact with PRMT5 (Figure 4E). Furthermore, in colorectal cancer cells with double knockout of endogenous AKT1 and AKT2, the ability of PRMT5 to activate the AKT/mTOR signaling pathway was rescued when AKT1 expression was restored with wild‐type AKT1 but not the AKT1 R391K mutant (which contains a mutation blocking its PRMT5‐mediated methylation ^[^ ^27^ ^]^) (Figure 4I,J; Figure S7B–D, Supporting Information). These results suggested that PRMT5 methylated AKT1 at R391 to activate the AKT/mTOR signaling pathway. In addition, AKT3 was undetectable in colorectal cancer cells ^[^ ^42^ ^]^ (Figure S7E, Supporting Information).

### 2.5 PRMT5 Methylates AKT1 at R391 to Promote Colorectal Cancer Liver Metastasis

**Figure 5:**
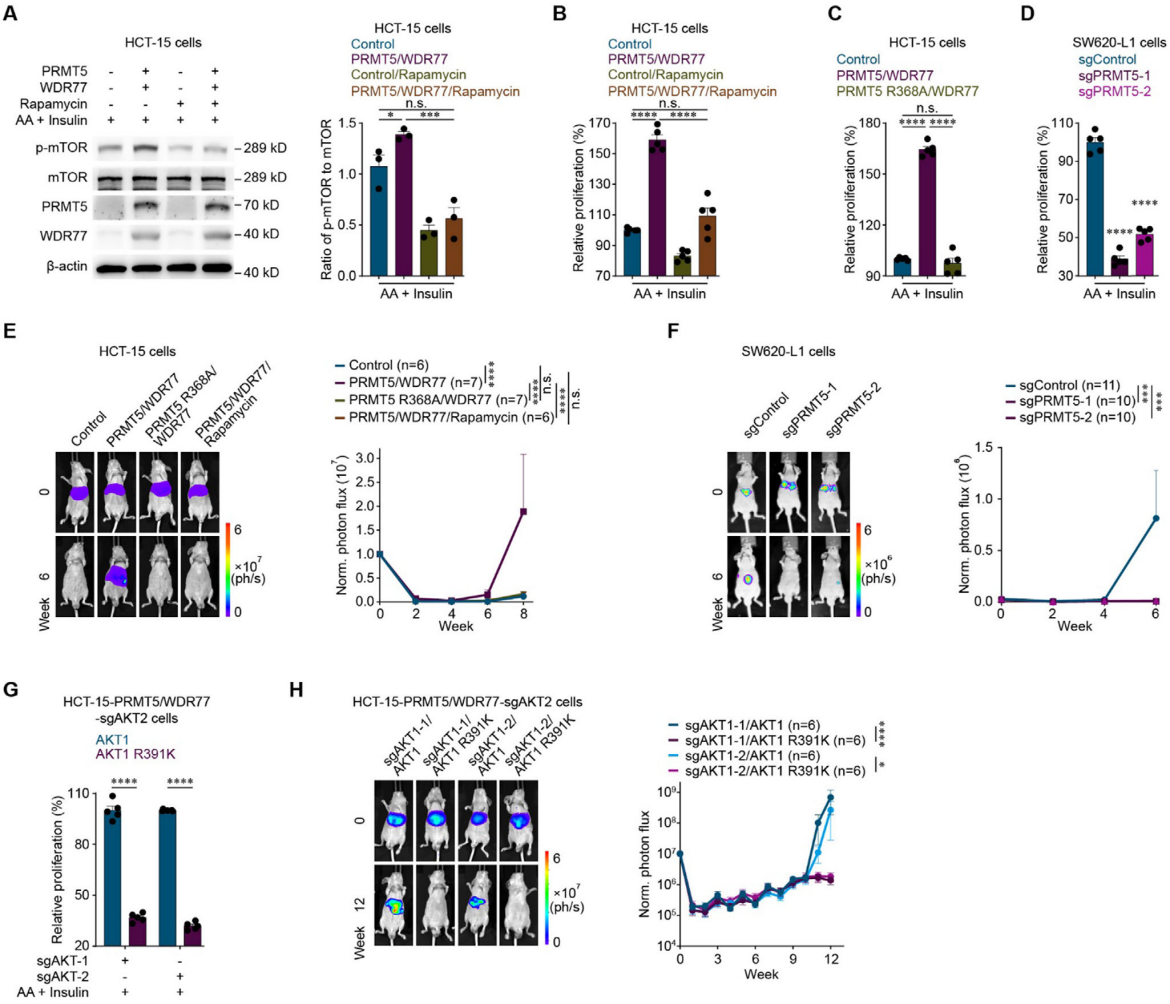
PRMT5 methylates AKT1 at R391 to promote colorectal cancer liver metastasis. A) Representative western blots (left) and quantification of p‐mTOR and mTOR levels (right) in Control or PRMT5/WDR77 HCT‐15 cells cultured with or without 100 nм rapamycin for 24 h in combination with 200 μм AA for 15 min and 800 nм insulin for 10 min. Three independent experiments were performed. B) In vitro proliferation assay of Control or PRMT5/WDR77 HCT‐15 cells (1000 cells) cultured with or without 100 nм rapamycin combined with 200 μм AA and 800 nм insulin for 24 h. C) In vitro proliferation assay of Control, PRMT5/WDR77 or PRMT5 R368A/WDR77 HCT‐15 cells (1000 cells) cultured with 200 μм AA and 800 nм insulin for 24 h. D) In vitro proliferation assay of sgControl, sgPRMT5‐1 or sgPRMT5‐2 SW620‐L1 cells (3000 cells) cultured with 200 μм AA and 800 nм insulin for 24 h. E) Bioluminescence imaging results (left) and quantification of liver metastases (right) in BALB/c nude mice implanted with Control, PRMT5/WDR77 or PRMT5 R368A/WDR77 HCT‐15 cells (3 × 10^6^ cells) via intrasplenic injection. Rapamycin (2 mg k^−1^g) was administered intraperitoneally every two days from the day cancer cell injection (day 0) to the experimental endpoint. The *n*‐ values denote the number of mice per group. F) Bioluminescence imaging results (left) and quantification of liver metastases (right) in BALB/c nude mice implanted with sgControl, sgPRMT5‐1 or sgPRMT5‐2 SW620‐L1 cells (1 × 10^6^ cells) via intrasplenic injection. The *n*‐ values denote the number of mice per group. G) In vitro proliferation assay of PRMT5/WDR77/sgAKT1‐1/sgAKT2/AKT1, PRMT5/WDR77/sgAKT1‐2/sgAKT2/AKT1HCT‐15, PRMT5/WDR77/sgAKT1‐1/sgAKT2/AKT1 R391K or PRMT5/WDR77/sgAKT1‐2/sgAKT2/AKT1 R391K HCT‐15 cells (1000 cells) cultured with 200 μм AA and 800 nм insulin for 24 h. H) Bioluminescence imaging results (left) and quantification of liver metastases (right) in BALB/c nude mice implanted with PRMT5/WDR77/sgAKT1‐1/sgAKT2/AKT1, PRMT5/WDR77/sgAKT1‐2/sgAKT2/AKT1HCT‐15, PRMT5/WDR77/sgAKT1‐1/sgAKT2/AKT1 R391K or PRMT5/WDR77/sgAKT1‐2/sgAKT2/AKT1 R391K HCT‐15 cells (3 × 10^6^ cells) via intrasplenic injection. The *n*‐ values denote the number of mice per group. Five independent experiments were performed (B‐D and G).The data are presented as the mean ± s.e.m. values. *P*‐ values were determined by unpaired one‐way ANOVA with uncorrected Fisher's LSD test (A‐D), or unpaired two‐way ANOVA with uncorrected Fisher's LSD test (E‐H). * *P* < 0.05; *** *P* < 0.001; **** *P* < 0.0001; n.s., not significant.

Consistent with published studies, our study demonstrated that PRMT5 methylates AKT1 at R391 to activate the AKT/mTOR signaling pathway in colorectal cancer cells. Here, we found that the overexpression of PRMT5 increased the proliferation of colorectal cancer cells (**Figure** **5**A,B) and that the effect of PRMT5 on colorectal cancer cell proliferation was reversed by treatment with rapamycin, a selective mTOR inhibitor ^[^ ^43^ ^]^ that suppresses the AKT/mTOR signaling pathway (Figure 5A,B). These results suggested that PRMT5 increased the colorectal cancer cell proliferation in an AKT/mTOR signaling pathway‐dependent manner. In addition, the overexpression of PRMT5 R368A (a methyltransferase‐inactive mutant of PRMT5) failed to increase the proliferation of colorectal cancer cells (Figure 5C). Moreover, PRMT5 deficiency, as well as the inhibition of PRMT5 by EPZ015666, decreased the proliferation of colorectal cancer cells (Figure 5D; Figure S8, Supporting Information).

Furthermore, in vivo functional experiments revealed that overexpression of PRTM5 promoted colorectal cancer liver metastasis (Figure 5E), whereas the overexpression of the methyltransferase‐inactive PRMT5 R368A mutant did not affect liver metastasis, and the ability of PRMT5 to promote metastasis was reversed by rapamycin treatment (Figure 5E). Similarly, PRMT5 knockout prevented colorectal cancer liver metastasis (Figure 5F). Moreover, in colorectal cancer cells with double knockout of endogenous AKT1 and AKT2, the ability of PRMT5 to increase cell proliferation and promote colorectal cancer liver metastasis was rescued when AKT1 expression was restored with wild‐type AKT1 but not the AKT1 R391K mutant (which contains a mutation blocking its PRMT5‐mediated methylation ^[^ ^27^ ^]^) (Figure 5G,H).

These results suggested that PRMT5‐mediated activation of the AKT/mTOR signaling pathway increased cancer cell proliferation to enhance colonization, in turn promoting colorectal cancer liver metastasis, and that this effect was dependent on the methyltransferase activity of PRMT5.

### 2.6 NEDD4L Promotes PRMT5 Degradation to Inhibit the AKT/mTOR Signaling Pathway and Prevent Colorectal Cancer Liver Metastasis

**Figure 6:**
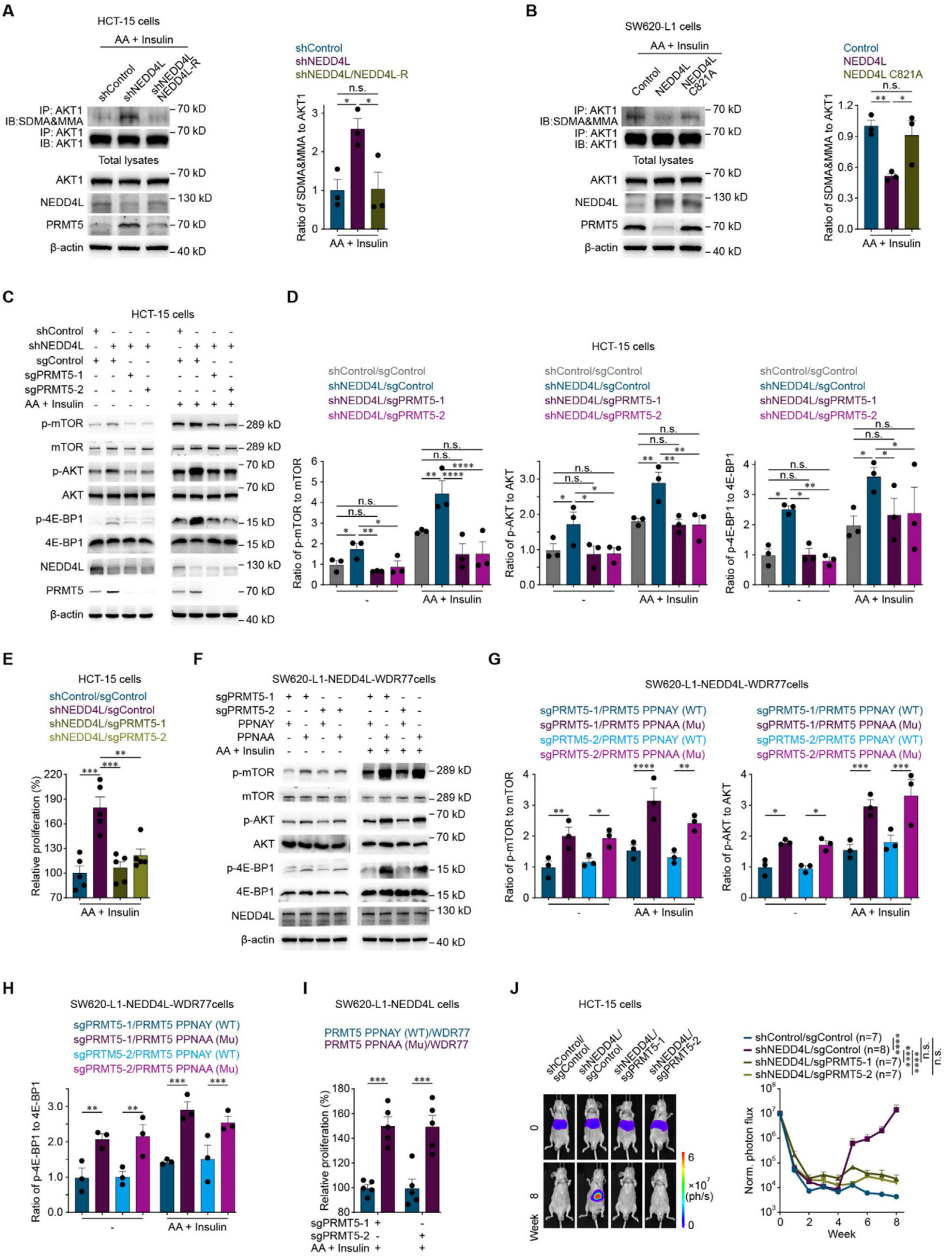
NEDD4L promotes PRMT5 degradation to inhibit the AKT/mTOR signaling pathway and prevent colorectal cancer liver metastasis. A) Representative western blots (left) and quantification of AKT1 methylarginine and AKT1 levels (right) in shControl, shNEDD4L or shNEDD4L/NEDD4L‐R HCT‐15 cells incubated with 200 μм AA for 15 min and 800 nм insulin for 10 min. B) Representative western blots (left) and quantification of AKT1 methylarginine and AKT1 levels (right) in Control, NEDD4L or NEDD4L C821A SW620‐L1 cells induced with 2 µg mL^−1^ doxycycline for 24 h and incubated with 200 μм AA for 15 min and 800 nм insulin for 10 min. C,D) Representative western blots (C) and quantification of p‐mTOR, mTOR, p‐AKT, AKT, p‐4E‐BP1 and 4E‐BP1 levels (D) in control HCT‐15 cells (shControl/sgControl), HCT‐15 cells with NEDD4L knockdown (shNEDD4L/sgControl), and HCT‐15 cells with NEDD4L knockdown in combination with the knockout of PRMT5 (shNEDD4L/sgPRMT5‐1 or shNEDD4L/sgPRMT5‐2) incubated with or without 200 μм AA for 15 min and 800 nм insulin for 10 min. E) In vitro proliferation assay of shControl/sgControl, shNEDD4L/sgControl, shNEDD4L/sgPRMT5‐1 or shNEDD4L/sgPRMT5‐2 HCT‐15 cells (1000 cells) cultured with 200 μм AA and 800 nм insulin for 24 h. Five independent experiments were performed. F–H) Representative western blots (F) and quantification of the p‐mTOR, mTOR, p‐AKT, AKT, p‐4E‐BP1 and 4E‐BP1 levels (G, H) in SW620‐L1 cells overexpressing NEDD4L and WDR77 (NEDD4L‐WDR77) in combination with the knockout of endogenous PRMT5 (sgPRMT5‐1 or sgPRMT5‐2) and overexpression of wild‐type PRMT5 (PPNAY) or mutant PRMT5 (PPNAA) incubated with or without 200 μм AA for 15 min and 800 nм insulin for 10 min. NEDD4L overexpression in cancer cells was induced with 2 µg mL^−1^ doxycycline for 24 h. I) In vitro proliferation assay of PPNAY/sgPRMT5‐1, PPNAA/sgPRMT5‐1, PPNAY/sgPRMT5‐2 or PPNAA/sgPRMT5‐2 SW620‐L1‐NEDD4L‐WDR77 cells (3000 cells) cultured with 200 μм AA and 800 nм insulin for 24 h. Five independent experiments were performed. J) Bioluminescence imaging results(left) and quantification of liver metastases (right) in BALB/c nude mice implanted with shControl/sgControl, shNEDD4L/sgControl, shNEDD4L/sgPRMT5‐1 or shNEDD4L/sgPRMT5‐2 HCT‐15 cells (3 × 10^6^ cells) via intrasplenic injection. The *n*‐ values denote the number of mice per group. Three independent experiments were performed (A‐D and F). The data are presented as the mean ± s.e.m. values. *P*‐ values were determined by unpaired one‐way ANOVA with uncorrected Fisher's LSD test (A, B, and E), or unpaired two‐way ANOVA with uncorrected Fisher's LSD test (D, E and G‐J). * *P* < 0.05; ** *P* < 0.01; *** *P* < 0.001; **** *P* < 0.0001; n.s., not significant.

As shown above, NEDD4L promotes PRMT5 degradation, and PRMT5 methylates AKT1 to activate the AKT/mTOR signaling pathway. Therefore, whether NEDD4L inhibits the AKT/mTOR signaling pathway by promoting PRMT5 degradation to attenuate the arginine methylation of AKT1 was examined. NEDD4L knockdown stabilized PRMT5, resulting in the arginine methylation of AKT1, whereas the overexpression of NEDD4L‐R in NEDD4L‐knockdown colorectal cancer cells promoted PRMT5 degradation to attenuate AKT1 methylarginine levels (**Figure** **6**A). In contrast, the overexpression of wild‐type NEDD4L but not the E3 ligase activity‐dead NEDD4L C821A mutant promoted PRMT5 degradation to attenuate AKT1 methylarginine levels (Figure 6B), suggesting that the function of NEDD4L in the arginine methylation of AKT1 depends on its E3 ligase activity.

Moreover, when PRMT5 was knocked out (Figure 6C–E) or when the PRMT5 PPNAA mutant, whose degradation cannot be mediated by NEDD4L, was overexpressed (Figure 6F–I), NEDD4L failed to inhibit the AKT/mTOR signaling pathway and had no effect on colorectal cancer cell proliferation. Furthermore, in vivo functional experiments revealed that when PRMT5 was knocked out, NEDD4L knockdown failed to promote colorectal cancer liver metastasis (Figure 6J). These results suggested that NEDD4L promoting PRMT5 degradation to inhibit the AKT/mTOR signaling pathway decreases colorectal cancer cell proliferation, resulting in the suppression of colonization, and ultimately the prevention of colorectal cancer liver metastasis.

**Figure 7:**
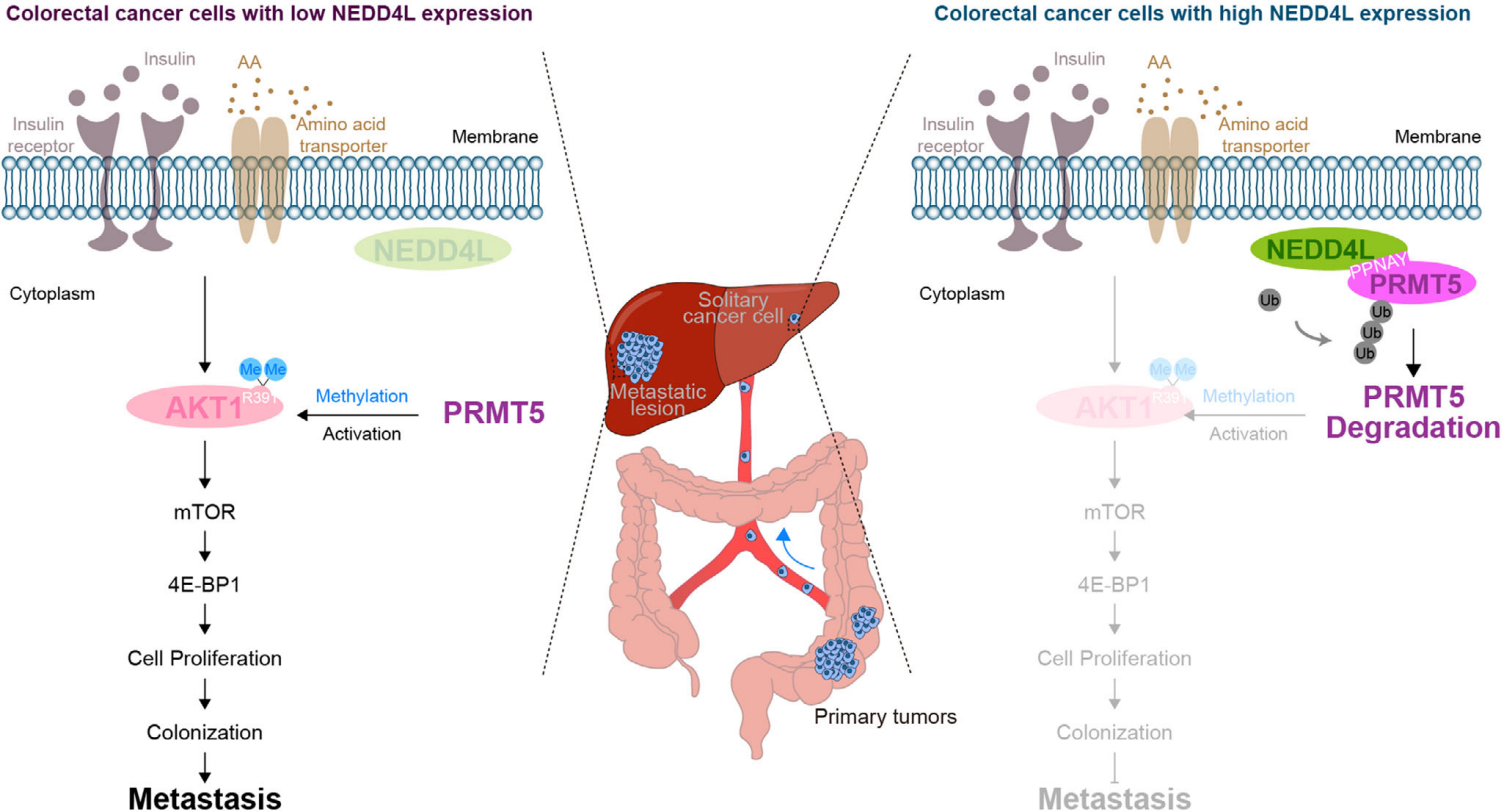
The mechanism by which NEDD4L prevents colorectal cancer liver metastasis. In colorectal cancer cells with high NEDD4L expression, the E3 ligase NEDD4L binds to the PPNAY motif in PRMT5 resulting in the ubiquitination and degradation of PRMT5. PRMT5 degradation attenuates the methylation of an arginine residue in AKT1 to inhibit the AKT/mTOR signaling pathway, consequently decreasing colorectal cancer cell proliferation to suppress colonization and ultimately prevent colorectal cancer liver metastasis. Conversely, in colorectal cancer cells with low expression of NEDD4L, the level of NEDD4L is not sufficient to induce the ubiquitination and degradation of PRMT5, resulting in an increase in AKT1 methylarginine levels to activate the AKT/mTOR signaling pathway. The activation of the AKT/mTOR signaling pathway increases colorectal cancer cell proliferation to enhance colonization and ultimately promotes colorectal cancer liver metastasis.

Collectively, our findings support the hypothesis that in liver‐metastatic colorectal cancer cells, NEDD4L binds to the PPNAY motif in PRMT5, resulting in the ubiquitination and degradation of PRMT5. PRMT5 degradation attenuates the methylation of an arginine residue in AKT1 to inhibit the AKT/mTOR signaling pathway. This effect decreases colorectal cancer cell proliferation to suppress colonization and ultimately prevents colorectal cancer liver metastasis (**Figure** **7**).

## 3 Discussion

Recently, various reports have shown that the dysfunctional activity of E3 ligases is associated with cancer.^[^ ^6^, ^7^, ^8^ ^]^ Since E3 ligases play a critical role in the specific recognition of substrate proteins,^[^ ^4^, ^5^ ^]^ targeting their active sites or interrupting the interactions between E3 ligases and their substrate proteins is a good approach for developing anticancer drugs with fewer side effects than the currently available drugs.^[^ ^44^ ^]^ To date, multiple drugs targeting E3 ligases have been approved by the FDA or evaluated in phase I/II clinical trials, such as thalidomide which suppresses CRBN activity (FDA‐approved for the treatment of multiple myeloma), and RG7112, which targets MDM2 to increase the p53 level (evaluated in a phase I clinical trial for hematological malignancies).^[^ ^45^ ^]^ Although liver metastasis is a major cause of death of patients with colorectal cancer,^[^ ^2^ ^]^ no drugs that target E3 ligases are available for the treatment of colorectal cancer liver metastasis.

Thus, we used an shRNA library targeting 156 E3 ubiquitin ligases to perform an in vivo loss‐of‐function screen of a human colorectal cancer cell line in a mouse model of colorectal cancer liver metastasis to identify the key E3 ligase involved in colorectal cancer liver metastasis. Through this screen, five E3 ligases were identified: NEDD4L, BMI1, SMURF1, AREL1 and FBXW2. NEDD4L was identified in two independent liver metastatic lesions derived from two different mice, and the liver metastasis incidence was 66.67% (2 of 3 mice) for NEDD4L‐knockdown HCT‐15 cells, whereas it was 33.33% (1 of 3 mice) for BMI‐, SMURF1‐, AREL1‐, and FBXW2‐knockdown HCT‐15 cells. These results suggested that NEDD4L exhibited the strongest metastasis‐inhibiting function (Figure S1A, Supporting Information). SMURF1 is a member of the NEDD4 family. The incidence of liver metastasis in SMURF1‐knockdown HCT‐15 cells was lower than that in NEDD4L‐knockdown HCT‐15 cells (Figure S1A, Supporting Information). Additionally, SMURF1 functions as a tumor suppressor by inducing the ubiquitination and degradation of TRIB2, AXIN1, MCAM and SRSF5.^[^ ^46^ ^]^ Thus, we speculate that SMURF1 might prevent colorectal cancer liver metastasis through mechanism other than NEDD4L‐mediated PRMT5 degradation. ITCH, HECW1, HECW2, NEDD4, WWP1, WWP2, and SMURF2, members of the NEDD4 family, were included in the shRNA library and were not identified in the screen. Moreover, published studies have revealed that these genes promote colorectal cancer progression.^[^ ^46^, ^47^, ^48^, ^49^, ^50^, ^51^, ^52^ ^]^ Combining the screening results with those of published studies, these findings suggested that these genes might promote colorectal cancer liver metastasis.

In colorectal cancer, NEDD4L has been reported to have diverse ubiquitination substrates, such as STK35, YBX1, and Wnt3.^[^ ^15^, ^18^, ^19^ ^]^ Previous studies have shown only the suppressive role of NEDD4L and its substrates in primary colorectal cancer.^[^ ^12^, ^14^, ^18^, ^19^, ^22^ ^]^ In this study, we focused on metastatic colonization in the liver, which is the rate‐limiting step of the metastatic cascade.^[^ ^32^ ^]^ We revealed that NEDD4L prevents colorectal cancer liver metastasis by inhibiting the proliferation of colorectal cancer cells. In this study, we not only revealed that NEDD4L prevented colorectal cancer liver metastasis but also identified a novel mechanism by which NEDD4L affected colorectal cancer liver metastasis. These results suggested that the E3 ligase NEDD4L could be exploited therapeutically for colorectal cancer liver metastasis. However, the use of only one NEDD4L shRNA in our study is one limitaion of our study. The shRNA library used for the screen contained five NEDD4L shRNAs. One shRNA (shNEDD4L, ID: TRCN0000000905) was identified in two independent liver metastatic lesions derived from two different mice, and had the highest knockdown efficiency (Figure 1B,C; Figure S1A,B, Supporting Information). Moreover, we did not find any published shRNAs (different from the five shRNAs in the library) whose knockdown efficiency was similar to that of shNEDD4L (TRCN0000000905) in our system. Therefore, as a method to avoid the off‐target effect of a single shRNA, NEDD4L‐R, which is resistant to shNEDD4L (TRCN0000000905) targeting, was used to restore NEDD4L expression in NEDD4L‐knockdown colorectal cancer cells.

Notably, we discovered that PRMT5 is a substrate protein of NEDD4L. Accumulating evidence indicates that PRMT5 functions as an oncogene in colorectal cancer and that its cancer‐promoting function depends on its methyltransferase activity,^[^ ^26^, ^27^, ^28^ ^]^ suggesting that PRMT5 is a potential target for cancer therapy. Indeed, different types of PRMT5 inhibitors have been developed for cancer treatment and are categorized into 5 distinct types: SAM‐competitive (JNJ64619178), substrate‐competitive (GSK3326595 and EPZ015666), MTA‐cooperative (AMG193 and TNG908), allosteric, and PRMT5‐adaptor protein‒protein interaction inhibitors.^[^ ^53^ ^]^ Currently, almost all the PRMT5 inhibitors under development have been designed to inhibit PRMT5 methyltransferase activity or disrupt the interactions between PRMT5 and its target proteins. This study revealed not only that the ubiquitin‒proteasome system regulates PRMT5 stability but also that the E3 ligase NEDD4L mediates the ubiquitination and degradation of PRMT5. These findings may provide a new strategy for PRMT5 degradation that differs from the traditional strategies designed to inhibit PRMT5‐mediated methylation of arginine.

Published studies have reported that the ubiquitylation and degradation of PRMT5 are regulated by multiple lysine residues, such as K227, K240K302, K329, K333, K343, K354, K380, and K387, in PRMT5.^[^ ^29^, ^30^ ^]^ Given that NEDD4L also mediates the ubiquitination and degradation of PRMT5, we hypothesize that the sites where NEDD4L ubiquitinates PRMT5 might be included in these lysine residues. Moreover, previous studies have shown that UbcH5B is the E2 enzyme that coordinates the ubiquitination mediated by NEDD4.^[^ ^54^ ^]^ and that NEDD4L ubiquitinates and degrades its substrates through K48‐linked ubiquitin chains.^[^ ^55^, ^56^ ^]^ Furthermore, the ubiquitination and degradation of PRMT5 also occur through K48‐linked ubiquitin chains.^[^ ^29^ ^]^ Considering this evidence, we speculate that NEDD4L might ubiquitinate and degrade PRMT5 through K48‐linked ubiquitination chains and that UbcH5B is a potential E2 enzymes that coordinates this process.

The AKT/mTOR signaling pathway is one of the most frequently altered pathways in human cancers.^[^ ^57^, ^58^ ^]^ Cancer cell proliferation is regulated mainly through AKT‐mediated activation of the protein kinase mTORC1.^[^ ^59^ ^]^ Both the dysfunction of AKT regulators and genetic alterations in AKT cause overactivation of the AKT pathway in various human cancers.^[^ ^27^ ^]^ Posttranslational modifications of AKT, such as methylarginine, lysine modifications, and tyrosine phosphorylation, are important for AKT hyperactivation in cancers.^[^ ^59^ ^]^ Yin et al. substituted the arginine 391 residue (R391) of AKT1 for lysine (K), and subsequently determined the methylarginine level of AKT1 R391K mutant using an antibody. The result indicated that PRMT5 failed to methylate the AKT1‐R391K mutant. LC‒MS/MS was subsequently used to confirm that PRMT5 methylated AKT1 at R391.^[^ ^27^ ^]^ Based on these findings, in our study, an antibody was also used to determine the methylarginine level of the AKT1‐R391K mutant in colorectal cancer cells when AKT was activated. These results indicated that the AKT1 R391K mutant was not methylated by PRMT5, suggesting that PRMT5 methylates AKT1 at R391. Moreover, PRMT5‐mediated methylation of AKT1 at R391 increased cell proliferation to enhance colonization, in turn promoting colorectal cancer liver metastasis (Figure 5G,H). In addition, we have revealed that the methylation of R391 in AKT1 occurred in a NEDD4L‐dependent manner (Figure 6A,B). Although functional experiments showed that NEDD4L prevented colorectal cancer liver metastasis through PRMT5 degradation to attenuate the methylation of R391 in AKT1, we did not obtain direct evidence that NEDD4L mediated PRMT5 degradation to attenuate the methylation of R391 in AKT1.

Taken together, our findings reveal that NEDD4L binds to the PPNAY motif in PRMT5 to induce its ubiquitination and degradation. PRMT5 degradation attenuates the methylation of AKT1 to inhibit the AKT/mTOR signaling pathway. The effect of NEDD4L decreased cancer cell proliferation to suppress colonization, and ultimately prevent colorectal cancer liver metastasis.

## 4 Conclusion

In this study, we revealed that NEDD4L prevented colorectal cancer liver metastasis and that the metastasis‐inhibiting function of NEDD4L depended on its E3 ubiquitin ligase activity. We discovered that ubiquitin–proteasome system regulated PRMT5 stability and that PRMT5 was a substrate protein of NEDD4L. Mechanistic studies revealed that NEDD4L bound to the PPNAY motif in PRMT5 and ubiquitinated PRMT5 to promote its degradation. PRMT5 degradation attenuates the methylation of an arginine residue in AKT1. The decrease in the methylarginine level of AKT1 inhibited the AKT/mTOR signaling pathway, consequently decreasing colorectal cancer cell proliferation and resulting in the suppression of colonization. These results may provide a potential target for colorectal cancer liver metastasis therapy.

## 5 Experimental Section

### In Vivo Loss‐Of‐Function Screen of E3 Ligases Involved in Colorectal Cancer Liver Metastasis

A total of 794 shRNAs targeting 156 cancer‐related E3 ubiquitin ligases (3‐9 shRNAs per ligase, Table S1, Supporting Information) were sellected to construct an shRNA library for an in vivo loss‐of‐function screen. The sequences of the shRNAs were obtained from Sigma–Aldrich (USA). The library was subdivided into six subpools. HCT‐15 human colorectal cancer cells stably expressing tdTomato‐luciferase were transduced independently with each of the six subpools at a multiplicity of infection (MOI) of 3:1, an MOI at which more than 90% of the HCT‐15 cells were successfully transduced with the subpools. The transduced HCT‐15 cells (3 × 10^6^ cells/100 µL of PBS) were subsequently implanted into BALB/c nude mice via intrasplenic injection. In the screen, 20 mice (shControl: 2 mice, and shPools A–F: 3 mice for each pool) were injected. Eight weeks after the injection, macroscopic metastatic lesions in the liver (visible to the naked eye) were harvested and minced to isolate cancer cells. The clonogenic cancer cells were expanded in medium supplemented with puromycin, and the genomic DNA of the cancer cells was extracted. The resident shRNA‐encoding sequences in the genomic DNA were amplified with primers (Table S3, Supporting Information), the PCR products were subsequently cloned and inserted into a TA cloning vector, and at least 15 independent clones were analyzed via Sanger sequencing. Moreover, the mRNA levels of the shRNAs targeting E3 ubiquitin ligases were measured by qPCR to assess the on‐target knockdown efficiency of each shRNA.

### Cell Lines

The HCT‐15 (K‐Ras G13D, Male, CCL‐225, RRID: CVCL_0292), SW480 (K‐Ras G12V/p53 R273H, Male, CCL‐228, RRID: CVCL_0546), LS 174T (K‐Ras G12D, Female, CL‐188, RRID: CVCL_1384), and COLO 320DM (p53 R248 W, Female, CCL‐220, RRID: CVCL_0219) human colorectal cancer cell lines were originally obtained from the American Type Culture Collection (ATCC, USA). The CT26 (K‐Ras G12D, Female, CRL‐2638, RRID: CVCL_7254) mouse colorectal cancer cell line was originally obtained from the ATCC. The SW620‐L1 (K‐Ras G12V/p53 R273H, Male) human colorectal cancer cell line was a kind gift from Dr. Filippo G. Giancotti (Columbia University, USA). The 293FT (R70007, Female, RRID: CVCL_6911) cell line was purchased from Life Technologies. HCT‐15 and CT26 cells were cultured in RPMI‐1640 medium (31800‐089, Thermo Fisher Scientific, USA) supplemented with 10% fetal bovine serum (FBS; A0500‐3010, Cegrogen, Germany), 2 mм L‐glutamine (21051‐024, Thermo Fisher Scientific), and 100 U mL^−1^ penicillin/0.1 mg mL^−1^ streptomycin (P/S; C0222, Beyotime Biotechnology, China). SW480 and SW620‐L1 cells were cultured in DMEM/F12 medium (12500‐062, Thermo Fisher Scientific) supplemented with 10% FBS, 2 mм L‐glutamine, P/S and 100 μм MEM nonessential amino acids solution (NEAA; 11140050, Thermo Fisher Scientific). LS 174T, COLO 320DM and 293FT cells were cultured in DMEM‐HG (12100‐061, Thermo Fisher Scientific) supplemented with 10% FBS, 2 mм L‐glutamine and P/S. For bioluminescent tracking, cell lines were transduced with a lentiviral vector encoding tdTomato and firefly luciferase, and tdTomato‐positive cells were isolated via FACS. The HCT‐15, SW620‐L1 and CT26 cells used in this study stably expressed both tdTomato and firefly luciferase. All human cell lines were authenticated by short tandem repeat (STR) profiling performed by the Bio‐Research Innovation Center Suzhou, SIBCB, CAS, and were routinely tested for mycoplasma contamination.

### Mice

The mice were housed under specific pathogen‐free (SPF) conditions in the animal facility of Tongji University. All animal experiments were approved by the Institutional Animal Care and Use Committee of Tongji University (approval number: TJBA01020104). BALB/c nude mice and BALB/c mice were purchased from Beijing Vital River Laboratory Animal Technology (Beijing, China). HCT‐15 cells, SW620‐L1 cells, and their derivatives were used to establish xenograft models in 5‐ to 7‐week‐old male BALB/c nude mice. CT‐26 cells and their derivatives were used to establish isograft models in 5‐ to 7‐week‐old male syngeneic BALB/c mice.

### Bioluminescence Imaging

Mice were anesthetized and injected retro‐orbitally with 1.5 mg of D‐luciferin (LUCK‐1G, Gold Biotechnology, USA) at the indicated times. The animals were imaged in a NightOWL II LB 983 chamber (BertholdTechnologies, Bad Wildbad, Germany), AniView 100 (Biolight Biotechnology, China) or IVIS Lumina XRMS Series III (PerkinElmer, USA) within 5 min after the D‐luciferin injection, and the data were recorded with Indigo software (BertholdTechnologies, Bad Wildbad, Germany), AniView software (Biolight Biotechnology, China) or Live Image software (PerkinElmer, USA) to measure liver metastasis. Photon flux was calculated in a circular region of interest (ROI) encompassing the liver of each mouse.

### Establishment of Mouse Models of Colorectal Cancer Liver Metastasis via Intrasplenic Injection

Cancer cells stably expressing tdTomato and firefly luciferase were harvested by trypsinization, washed twice with PBS, resuspended at 3 × 10^6^ (transduced shControl/Control, shNEDD4L/Control, shNEDD4L/NEDD4L‐R, Control, PRMT5/WDR77 or PRMT5 R368A/WDR77 HCT‐15 cells), 3 × 10^6^ (transduced shControl/sgControl, shNEDD4L/sgControl, shNEDD4L/sgPRMT5‐1, shNEDD4L/ sgPRMT5‐2, sgAKT1‐1/sgAKT2/AKT1/PRMT5/WDR77, sgAKT1‐1/sgAKT2/AKT1 R391K/PRMT5/WDR77, sgAKT1‐2/sgAKT2/AKT1/PRMT5/WDR77, sgAKT1‐2/ sgAKT2/AKT1 R391K/PRMT5/WDR77 HCT‐15‐Cas9 cells), 1 × 10^6^ (transduced inducible Control, NEDD4L, NEDD4L C821A or NEDD4L R776Q SW620‐L1 cells), 1 × 10^6^ (transduced sgControl, sgPRMT5‐1 or sgPRMT5‐2 SW620‐L1‐Cas9 cells), or 3 × 10^5^ (transduced induced Control or NEDD4L CT26 cells) cells in 100 µL of PBS, and then injected into the spleens of the mice. The spleens were excised 2 min after the cancer cell injection. Bioluminescence imaging was used to verify the success of the injection after surgery and to monitor metastatic outgrowth. Doxycycline (D9891, Sigma; 100 µg doxycycline in 100 µL of PBS) was administered intraperitoneally on the day of cancer cell injection (day 0) and then administered orally (400 ppm in chow combined with 2 mg mL^−1^ in water) from day 0 to the experimental endpoint to induce the overexpression of NEDD4L or its mutants in SW620‐L1 cells. Rapamycin (GC15031, GlpBio, USA; 2 mg kg^−1^) was administered intraperitoneally every two days from the day of the cancer cell injection (day 0) to the experimental endpoint to inhibit the mTOR signaling pathway in vivo.

### Immunostaining

Cancer cells stably expressing tdTomato and firefly luciferase were harvested by trypsinization, washed twice with PBS, resuspended 1 × 10^6^ SW620‐L1 cells (transduced inducible Control, NEDD4L, shControl or shNEDD4L) in 100 µL of PBS, and then injected into the spleens of the mice. The spleens were excised 2 min after cancer cell injection. Doxycycline (100 µg doxycycline in 100 µL of PBS) was administered intraperitoneally on day 7 after the cancer cell injection and then administered orally (400 ppm in chow combined with 2 mg mL^−1^ in water) from day 7 to day 13 after the cancer cell injection to induce NEDD4L overexpression in SW620‐L1 cells. The Mice were sacrificed and perfused with PBS through the inferior vena cava on day 13 (inducible NEDD4L overexpressing and Control cells) or day 14 (shControl and shNEDD4L cells) after the cancer cell injection. The entire liver was divided into 7 parts based on the liver lobes. The liver lobes were fixed with 4% paraformaldehyde (PFA) overnight at 4 °C, dehydrated in 30% sucrose for 48 h, and embedded in optimal cutting temperature (OCT) compound. The entire liver lobe was serially sectioned (10 µm thick) with a Thermo Scientific Cryostar NX50, and immunofluorescence staining was performed. An anti‐tdTomato antibody was used with a Tyramide Signal Amplification Kit (B40931, Thermo Fisher Scientific) to visualize cancer cells, and staining with an anti‐Ki67 antibody was then performed to assess proliferation. Immunoreactions were detected with fluorescently labeled secondary antibodies. The sections were mounted with ProLong Gold Antifade Mountant with DAPI (P36931, Thermo Fisher Scientific) and imaged with a Carl Zeiss LSM900 confocal microscope. The number or area of metastatic lesions in the entire liver were counted at 40× magnification. For each metastatic lesion, three random sections were selected, the Ki67‐positive rate was determined by calculating the percentage of Ki67‐positive cells among the total population of tumor cells in each section, and the percentage of Ki67‐positive cells in each metastatic lesion was then calculated as the average percentage across the three sections. Since no metastatic lesions or cells were present in the liver of mice in the NEDD4L group, the percentage of Ki67‐positive cells was considered 0.

### CCK‐8 Assay

HCT‐15 cells transduced with shControl/Control, shNEDD4L/Control, or shNEDD4L/NEDD4L‐R (1000 cells) were seeded into 96‐well plates and cultured for 72 h. SW620‐L1 cells transduced with Control, NEDD4L, NEDD4L C821A, or NEDD4L R776Q (3000 cells) were seeded into 96‐well plates and cultured with 2 µg mL^−1^ doxycycline for 72 h. At the indicated time points, the CCK‐8 solution (CK04, Dojindo, Japan) was added to each well, and the plates were incubated for 1 h at 37 °C. The absorbance was measured at 450 nm with a spectrophotometer (Infinite M200 Pro, TECAN, Switzerland). HCT‐15 cells transduced with shControl/Control, shNEDD4L/Control, shNEDD4L/NEDD4L‐R, Control, PRMT5/WDR77, or PRMT5 R368A/WDR77 (1000 cells); HCT‐15‐Cas9 cells transduced with shControl/sgControl, shNEDD4L/sgControl, shNEDD4L/sgPRMT5‐1, shNEDD4L/sgPRMT5‐2, sgAKT1‐1/sgAKT2/AKT1/PRMT5/WDR77, sgAKT1‐1/sgAKT2/AKT1 R391K/PRMT5/ WDR77, sgAKT1‐2/sgAKT2/AKT1/PRMT5/WDR77, or sgAKT1‐2/sgAKT2/AKT1 R391K/PRMT5/WDR77 (1000 cells); SW620‐L1 cells transduced with Control, NEDD4L, NEDD4L C821A, or NEDD4LR776Q (3000 cells); or SW620‐L1‐Cas9 cells transduced with sgControl, sgPRMT5‐1, sgPRMT5‐2, NEDD4L/sgPRMT5‐1/PRMT5‐PPNAY, NEDD4L/sgPRMT5‐1/PRMT‐PPNAA, NEDD4L/sgPRMT5‐2/PRMT5‐PPNAY, or sgPRMT5‐2/NEDD4L/PRMT5‐PPNAA (3000 cells) were seeded into 96‐well plates and cultured for 48 h. The medium was replaced with FBS‐free RPMI‐1640 medium (for HCT‐15 cells) or DMEM/F12 medium (for SW620‐L1 cells), and the cancer cells were cultured for another 24 h. The medium was subsequently replaced with FBS‐free, amino acid‐free RPMI‐1640 medium (for HCT‐15 cells, YYX2009, Yuyan Biotechnology, China) or amino acid‐free DMEM/F12 medium (for SW620‐L1 cells, YYx02010, Yuyan Biotechnology) supplemented with 800 nм insulin (I9278, Sigma) and 200 μм NEAA, and the cancer cells were cultured for another 24 h. SW620‐L1 cells were cultured with 2 µg mL^−1^ doxycycline to induce the overexpression of NEDD4L or its mutants. HCT‐15 cells transduced with shControl, shNEDD4L, Control, PRMT5/WDR77 or PRMT5‐R368A/WDR77 were cultured with 100 nм rapamycin for 24 h to inhibit the mTOR signaling pathway. At the indicated time points, CCK‐8 solution was added to each well, and the plates were incubated for 2 h at 37 °C. The absorbance was measured at 450 nm with a spectrophotometer.

### Coimmunoprecipitation (Co‐IP) Assay

SW620‐L1 cells or HCT‐15 cells were seeded in a 10 cm dish and cultured for 48 h to confirm the interaction between endogenous PRMT5 and NEDD4L. The cells were lysed in co‐IP radioimmunoprecipitation assay (RIPA) lysis buffer (50 mм Tris‐HCl (pH 7.4), 150 mм NaCl, 1% NP‐40, and 0.25% sodium deoxycholate) supplemented with phenylmethylsulfonyl fluoride (PMSF; ST505, Beyotime Biotechnology) and a phosphatase inhibitor cocktail (B15001, Bimake, USA). Total lysates containing 1 mg of protein were incubated with 1 µg of a rabbit polyclonal anti‐PRMT5 antibody or a rabbit polyclonal anti‐NEDD4L antibody overnight at 4 °C and then incubated with 20 µL of Protein A/G agarose (22851, Thermo Fisher Scientific) for 3 h at 4 °C to immunoprecipitate PRMT5 or NEDD4L. SW620‐L1 cells were seeded in 10 cm dishes and cultured for 48 h to confirm the interaction between endogenous PRMT5 and AKT1. The medium was replaced with FBS‐free DMEM/F12, and the cells were cultured for 24 h. The medium was then replaced with FBS‐free, amino acid‐free DMEM/F12, and the cells were cultured for 50 min prior to incubation with 200 μм NEAA for 15 min and 800 nм insulin for 10 min. After treatment, the cells were lysed in co‐IP RIPA lysis buffer supplemented with PMSF and a phosphatase inhibitor cocktail. Total lysates containing 1 mg of protein were incubated with 1 µg of a rabbit polyclonal anti‐PRMT5 antibody overnight at 4 °C and then incubated with 20 µL of Protein A/G agarose for 3 h at 4 °C to immunoprecipitate PRMT5. 293FT cells transfected with control or Flag‐PRMT5/Myc‐WDR77 in combination with HA‐AKT, HA‐TSC2 or Myc‐Rheb were cultured for 48 h to confirm the interactions between exogenous Flag‐PRMT5 and AKT/mTOR signaling pathway‐related proteins (HA‐AKT, HA‐TSC2 and Myc‐Rheb). The medium was then replaced with FBS‐free DMEM‐HG, and the cells were cultured for another 24 h. Then, the medium was replaced with FBS‐free, amino acid‐free DMEM (CB000‐8001, Excell Bio, China), and the cells were cultured for 50 min prior to incubation with 200 μм NEAA for 15 min and 800 nм insulin for 10 min. After treatment, the cells were lysed in co‐IP RIPA lysis buffer supplemented with PMSF and a phosphatase inhibitor cocktail. Total lysates containing 1 mg of protein were incubated with 20 µL of anti‐FLAG M2 Affinity Gel (A2220, Sigma) for 2 h at 4 °C to immunoprecipitate Flag‐PRMT5. The immunoprecipitates and total lysates were then subjected to immunoblotting with the indicated antibodies (Table S4, Supporting Information).

### Mass Spectrometry

293FT cells were transiently transfected with the control plasmid or with Flag‐NEDD4L and HA‐ubiquitin to identify the substrate of NEDD4L. After 48 h, the cells were treated with 20 μм MG132 (GC10383, GlpBio) for 12 h and were lysed in lysis buffer (50 mм Tris‐HCl (pH 7.4), 150 mм NaCl, 1 mм EDTA, 1% glycerol, 1% Triton X‐100, and 0.1% sodium dodecyl sulfate (SDS)) supplemented with 1 mм Na_3_VO_4_, a phosphatase inhibitor cocktail, and protease inhibitors. The lysates were then incubated with anti‐FLAG M2 affinity gel for 2 h at 4 °C. After washing three times, the protein–gel mixture was eluted with Flag peptide (A6001, Apexbio, USA) for 30 min according to the manufacturer's protocols. For serial co‐IP, the immunoprecipitates obtained with the anti‐FLAG M2 affinity gel were incubated with the EZview Red Anti‐HA affinity gel (E6779, Sigma) for 12 h at 4 °C. After washing three times, the protein–gel mixture was subjected to elution with the HA peptide (A6004, Apexbio) for 30 min according to the manufacturer's protocols. The immunoprecipitates were separated on a NuPAGE 10% Bis‐Tris gel (NP0341BOX, Thermo Fisher Scientific) and visualized with EZBlue Gel Staining Reagent (G1041‐500ML, Sigma). The complete gel lanes were excised and subjected to electrospray ionization–liquid chromatography–tandem mass spectrometry (ESI‐LC‐MS/MS) analysis.

### Analysis of Protein Expression

Cancer cells were lysed in RIPA buffer (50 mм Tris‐HCl (pH 7.4), 150 mм NaCl, 1 mм EDTA, 1% Triton X‐100, 1% sodium deoxycholate, and 0.1% SDS) supplemented with PMSF and a phosphatase inhibitor cocktail, unless indicated otherwise. Protein concentrations were measured with an Enhanced BCA Protein Assay Kit (P0010, Beyotime). The total lysates were subjected to immunoblotting with the indicated antibodies (Table S4, Supporting Information). Protein expression was quantified with ImageJ.

### Analysis of Protein Methylarginine Levels

SW620‐L1‐Cas9 cells transduced with sgControl, sgPRMT5‐1, or sgPRMT5‐2; SW620‐L1 cells transduced with Control, NEDD4L, or NEDD4L C821A; HCT‐15‐Cas9 cells transduced with sgAKT1‐1/AKT1/PRMT5/WDR77, sgAKT1‐1/AKT1 R391K/PRMT5/WDR77, sgAKT1‐2/AKT1/PRMT5/WDR77, or sgAKT1‐2/AKT1 R391K/PRMT5/WDR77; HCT‐15 cells transduced with shControl/Control, shNEDD4L/Control, or shNEDD4L/NEDD4L‐R; and HCT‐15 cells transduced with Control, PRMT5/WDR77, or PRMT5 R368A/WDR77 (5 × 10^5^ cells) were seeded in a 10 cm dish and cultured for 48 h. The medium was replaced with FBS‐free DMEM/F12 (for SW620‐L1 cells) or RPMI‐1640 (for HCT‐15 cells), and the cells were cultured for 24 h. The medium was then replaced with FBS‐free, AA‐free DMEM/F12 (for SW620‐L1 cells) or RPMI‐1640 (for HCT‐15 cells), and the cells were cultured for 50 min prior to an incubation with 200 μм NEAA for 15 min and 800 nм insulin for 10 min. The overexpression of NEDD4L or NEDD4L C821A was induced in SW620‐L1 cells through culture with 2 µg mL^−1^ doxycycline. SW620‐L1 cells (5 × 10^5^ cells) were seeded in 10 cm dishes and cultured for 48 h. Then, the medium was replaced with FBS‐free DMEM/F12, and the cells were cultured with EPZ015666 (0, 0.31 or 1.25 μм) for 48 h. The medium was then replaced with FBS‐free, AA‐free DMEM/F12 for 50 min prior to an incubation with 200 μм NEAA for 15 min and 800 nм insulin for 10 min. After treatment, the cells were lysed in RIPA lysis buffer supplemented with PMSF and a phosphatase inhibitor cocktail. AKT1 was immunoprecipitated to analyze the AKT1 methylarginine level. Total lysates containing 1 mg of protein were incubated with 1 µg of a rabbit monoclonal anti‐AKT1 antibody overnight at 4 °C and were then incubated with 20 µL of Protein A/G agarose for 3 h at 4 °C. The pan‐AKT was immunoprecipitated by immunoprecipitating total AKT. Total lysates containing 1 mg of protein were incubated with 1 µg of the rabbit monoclonal anti‐AKT antibody overnight at 4 °C and were then incubated with 20 µL of Protein A/G agarose for 3 h at 4 °C. Immunoprecipitates and total lysates were subjected to immunoblotting with the indicated antibodies (Table S4, Supporting Information).

### Analysis of Protein Ubiquitination Levels

A total of 5 × 10^5^ SW620‐L1 cells (transduced with inducible Control, NEDD4L, NEDD4L C821A or NEDD4L R776Q), 5 × 10^5^ HCT‐15 cells (transduced with shControl/Control, shNEDD4L/Control or shNEDD4L/NEDD4L‐R), or 1 × 10^6^ 293FT cells (transfected with Control/Control/HA‐ubiquitin, Control/Myc‐PRMT5/HA‐ubiquitin, Flag‐NEDD4L/Myc‐PRMT5/HA‐ubiquitin, Flag‐NEDD4L C821A/Myc‐PRMT5/HA‐ubiquitin, or Flag‐NEDD4L R776Q/Myc‐PRMT5/HA‐ubiquitin) were seeded into 10 cm dishes and cultured for 48 h. Subsequently, 20 μм MG132 was added, and the cells were incubated for 12 h. After treatment, the cells were lysed in co‐IP RIPA lysis buffer supplemented with PMSF and phosphatase inhibitors. SW620‐L1 cells were cultured with 2 µg mL^−1^ doxycycline to induce the overexpression of NEDD4L or its mutants. Total lysates containing 1 mg of protein were incubated with 1 µg of a rabbit monoclonal anti‐PRMT5 antibody overnight at 4 °C and then incubated with 20 µL of Protein A/G Plus Agarose for 3 h at 4 °C to immunoprecipitate PRMT5. A total of 1 × 10^6^ 293FT cells transfected with Control/HA‐ubiquitin, NEDD4L/HA‐ubiquitin, NEDD4L C821A/HA‐ubiquitin or NEDD4L R776Q/HA‐ubiquitin were seeded into 10 cm dishes and cultured for 48 h. Subsequently, 20 μм MG132 was added, and the cells were incubated for 12 h. After treatment, the cells were lysed in co‐IP RIPA lysis buffer supplemented with PMSF and phosphatase inhibitors. Total lysates containing 1 mg of protein were incubated with 20 µL of Anti‐FLAG M2 Affinity Gel for 2 h at 4 °C to immunoprecipitate Flag‐NEDD4L. Immunoprecipitates and total lysates were subjected to immunoblotting with the indicated antibodies (Table S4, Supporting Information).

### Antibodies

All the antibodies and their applications are listed in Table S4 (Supporting Information).

### Analysis of mRNA Expression

Total RNA was extracted with an RNAprep Pure Cell/Bacteria Kit (DP430, TIANGEN, China) and reverse transcribed with a ReverTra Ace qPCR RT Kit (FSQ‐101, TOYOBO, Japan). An amount of cDNA corresponding to ≈10 ng of input RNA was used for each reaction. qPCR was performed with a TaqMan Gene Expression Assay (Applied Biosystems, USA) or 2 × SYBR Green qPCR Master Mix (Low ROX, B21702, Bimake). All expression levels were normalized to those of endogenous *ACTB* (β‐actin). All the experiments were performed on an Applied Biosystems 7500/7500 Fast instrument. The specific TaqMan gene expression assays and the sequences of the primers used to amplify the genes are listed in Table S3 (Supporting Information).

### Plasmids

Constructs encoding shRNAs against human NEDD4L (TRCN0000000905) and human PRMT5 (#1, TRCN0000107088 and #2, TRCN0000107086) were generated by cloning the corresponding shRNA sequences into the pLKO.1 vector. The cDNAs encoding full‐length human NEDD4L, PRMT5, WDR77, AKT, AKT1 and ubiquitin were cloned and sequenced. The pCDNA3.1‐HA‐TSC2 and pCDNA3.1‐Myc‐Rheb plasmids were kind gifts from Dr. Ping Wang (Tongji University, China). Flag‐NEDD4L, Flag‐NEDD4L C821A, and Flag‐NEDD4L R776Q were subcloned and inserted into the pCW lentiviral vector or pQCXIP (Clontech, Japan) retroviral vector and verified by sequencing. HA‐AKT, HA‐ubiquitin, Flag‐PRMT5, Myc‐WDR77, Myc‐PRMT5‐PPNAY, Myc‐PRMT5‐PANAY, Myc‐PRMT5‐PPNAA, Myc‐PRMT5‐PPNYY, Flag‐PRMT5‐2A‐Myc‐WDR77 and Flag‐PRMT5/R368A‐2A‐Myc‐WDR77 were subcloned and inserted into the pQCXIP (Clontech) retroviral vector and verified by sequencing. Flag‐NEDD4L‐R, Myc‐PRMT5, Myc‐PRMT5‐PPNAA, Flag‐PRMT5‐2A‐Myc‐WDR77 were subcloned and inserted into the pQCXIN (Clontech) retroviral vector and verified by sequencing. Flag‐AKT1 and Flag‐AKT1/R391K were subcloned and inserted into the pBABE‐Puro (Clontech) retroviral vector and verified by sequencing. The sgPRMT5‐1 and sgPRMT5‐2 sequences were cloned and inserted into the lentiGuide‐Puro lentiviral vector or lentiGuide‐EGFP lentiviral vector, the sgAKT1‐1 and sgAKT1‐2 sequences were cloned and inserted into the lentiGuide‐EGFP lentiviral vector, and the sgAKT2 sequence was cloned and inserted into the lentiGuide‐BFP vector to construct the single guide RNA (sgRNA) expression vector. All guide sequences are listed in Table S5 (Supporting Information). Populations of overexpression, knockdown, or knockout cells were used, and the overexpression, knockdown, or knockout efficiency was verified by western blotting.

### Matrigel Invasion Assay

HCT‐15 cells transduced with shControl or shNEDD4L (1000 cells) were cultured in RPMI‐1640 medium on the Matrigel‐coated (356237, Corning, USA; 100 µg/well) membranes of transwell cell culture inserts. After a 6‐h incubation in RPMI‐1640 supplemented with 10% FBS, the remaining cells and Matrigel in the upper compartment of the inserts were removed by wiping the upper side of the membrane with cotton swabs. The cells that invaded through the inserts were subsequently fixed with 4% PFA for 10 min at room temperature and stained with crystal violet (C0121, Beyotime). All invaded cells in each insert were counted at 400× magnification (3 inserts per group).

### Tumor Sphere Formation Assay

HCT‐15 cells transduced with shControl or shNEDD4L (1000 cells) were seeded in 24‐well ultralow‐attachment plates (Corning) and cultured for 7 days in serum‐free MEGM (Lonza, Switzerland) supplemented with B27 (1:50; Thermo Fisher Scientific), 20 ng mL^−1^ EGF (Thermo Fisher Scientific), 10 ng mL^−1^ bFGF (Thermo Fisher Scientific), and 2 µg mL^−1^ heparin (Sigma). All tumor spheres in each well were counted.

### Survival Analysis

The colorectal cancer dataset GSE17536, the signal intensity matrices calculated with MAS5 and the clinical information were downloaded from the Gene Expression Omnibus (GEO) website. The gene expression levels for each patient were log2 transformed and converted to z scores. The best probe to represent NEDD4L was selected with the R package “JetSet”. The RNA‐seq data matrices and clinical information of the patients represented in TCGA colorectal cancer (COAD+READ) datasets were downloaded from cBioPortal (https://www.cbioportal.org/datasets). The gene expression levels for each patient were log2 transformed and converted to z scores. Clinical information for each patient with an available survival time was collected for the survival analysis. The statistical significance of differences in survival was evaluated by performing a Kaplan‒Meier analysis with the log‐rank test (implemented with the “survdiff” function in the “survival” package in R). The data were plotted in Kaplan‒Meier survival curves.

### GSEA

GSEA was performed with the GSEA platform (GSEA v4.0.3). The proliferation‐related pathways were analyzed. The patients were classified by their NEDD4L expression level (high‐ and low‐ expression groups) for the survival analysis. The default values were used, except that the metric for ranking genes was set to “t test” and the permutation type was set to “gene set”.

### Analysis of NEDD4L mRNA Expression in Primary Tumors and Liver Metastatic Lesions from Patients with Colorectal Cancer

Five publicly available colorectal cancer microarray datasets (GSE10961, GSE18462, GSE28702, GSE40367, and GSE41568) in the GPL570 platform (Affymetrix Human Genome U133 Plus 2.0 Array) were downloaded from the GEO website. Gene expression was summarized by normalizing the data in each raw CEL file with the MAS5 algorithm in the R statistical environment (www.r‐project.org) using the Affy Bioconductor library. The primary tumor and liver metastatic lesions of the patients with colorectal cancer were unpaired. A publicly available microarray colorectal cancer dataset (GSE14297) was downloaded from the GEO website. In this dataset, the primary tumor and liver metastatic lesions of patients with colorectal cancer were paired.

### Statistical Analysis

The group sizes used for the in vivo and in vitro experiments were selected on based on intragroup variation. Data analyses and figure plotting were performed with GraphPad Prism (version 8.3.0). The numbers of replicates, independent experiments and statistical tests for each experiment are listed in the figure legends. The values are presented as the mean ± s.e.m, unless stated otherwise. For all analyses, a *P*‐ value of < 0.05 was considered to indicate statistical significance.

## Conflict of Interest

The authors declare no conflict of interest.

## Author Contributions

Z.D., X.S., J.M., Q.C., and Y.G. contributed equally and are the co‐first authors. Z.D., X.S., J.M., Q.C., and Y.G. designed, performed, and analyzed most experiments with assistance from R.C. X.S. performed the bioinformatic analyses. X.S. and H.G. wrote the manuscript with input from Z.D. H.Q. supported the study. The experiments supplied in revision manuscript were designed, supervised and funded by B. S. H.G. conceived, designed, interpreted, and supervised the study.

## Supporting information



Supporting Information

## Data Availability

Data sharing is not applicable to this article as no new data were created or analyzed in this study.
