# Supplementary material for: The E3 Ligase NEDD4L Prevents Colorectal Cancer Liver Metastasis via Degradation of PRMT5 to Inhibit the AKT/mTOR Signaling Pathway
Source: Adv Sci (Weinh). 2025 Apr 25;12(26):2504704. doi: 10.1002/advs.202504704 (PMC12244502; doi:10.1002/advs.202504704)
Supplement: Supplementary file 1 — Supporting Information [file ADVS-12-2504704-s001.pdf]

ADVANCED SCIENCE Open Access

Supporting Information

for *Adv. Sci.*, DOI 10.1002/advs.202504704

The E3 Ligase NEDD4L Prevents Colorectal Cancer Liver Metastasis via Degradation of PRMT5 to Inhibit the AKT/mTOR Signaling Pathway

Zhewen Dong, Xiaofei She, Junxian Ma, Qian Chen, Yaqun Gao, Ruiyan Chen, Huanlong Qin, Bing Shen* and Hua Gao**

Supporting Information

The E3 Ligase NEDD4L Prevents Colorectal Cancer Liver Metastasis via Degradation of PRMT5 to Inhibit the AKT/mTOR Signaling Pathway

Zhewen Dong, Xiaofei She, Junxian Ma, Qian Chen, Yaqun Gao, Ruiyan Chen, Huanlong Qin, Bing Shen*, Hua Gao**

A

Results of *in vivo* functional shRNA library targeting human E3 ubiquitin ligases screen.

Gene ID	Target Genes	shRNA ID	Sub-pool	No. of mets	Incidence of liver metastasis
**23327**	**NEDD4L: NEDD4 like E3 ubiquitin protein ligase**	**TRCN0000000905**	**shPool A**	**2 mets from 2 mice**	**66.67%**
648	BMI1: B lymphoma Mo-MLV insertion region	TRCN0000020158	shPool B	1 mets	33.33%
57154	SMURF1: SMAD specific E3 ubiquitin protein ligase 1	TRCN0000003472	shPool B	1 mets	33.33%
9870	AREL1: apoptosis resistant E3 ubiquitin protein ligase 1	TRCN0000004425	shPool D	1 mets	33.33%
26190	FBXW2: F-box and WD repeat domain containing 2	TRCN0000006549	shPool E	1 mets	33.33%

B

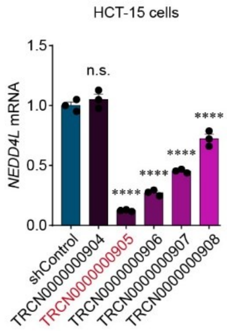

C

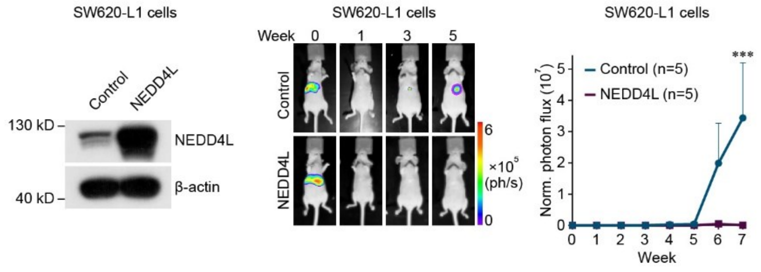

D

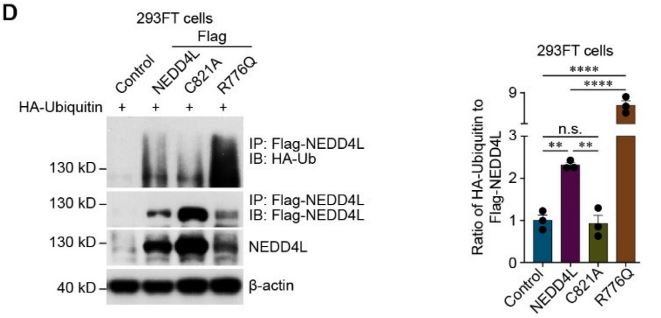

Figure S1. NEDD4L prevents colorectal cancer liver metastasis.

(A) Candidate human E3 ligases identified via *in vivo* functional screen.

(B) qPCR analysis of NEDD4L mRNA expression in shControl and NEDD4L-knockdown HCT-15 cells.

(C) Representative western blots showing NEDD4L expression in Control or NEDD4L SW620-L1 cells (left). Bioluminescence imaging results (middle) and quantification of liver metastases (right) in BALB/c nude mice implanted with Control or NEDD4L SW620-L1 cells (1×10^6 cells) via intrasplenic injection. The n- values denote the number of mice per group.

(D) Representative western blots (left) and qualification of Flag-NEDD4L ubiquitination and NEDD4L expression (right) in Control, NEDD4L, NEDD4L C821A or NEDD4L R776Q 293FT cells.

Three independent experiments were performed (B, D, and western blots in C). The data are presented as the mean ± s.e.m. values. *P*- values were determined by unpaired one-way ANOVA with uncorrected Fisher's LSD test (B, D), or unpaired two-way ANOVA with uncorrected Fisher's LSD test (C). ** $P < 0.01$; *** $P < 0.001$; **** $P < 0.0001$; n.s., not significant.

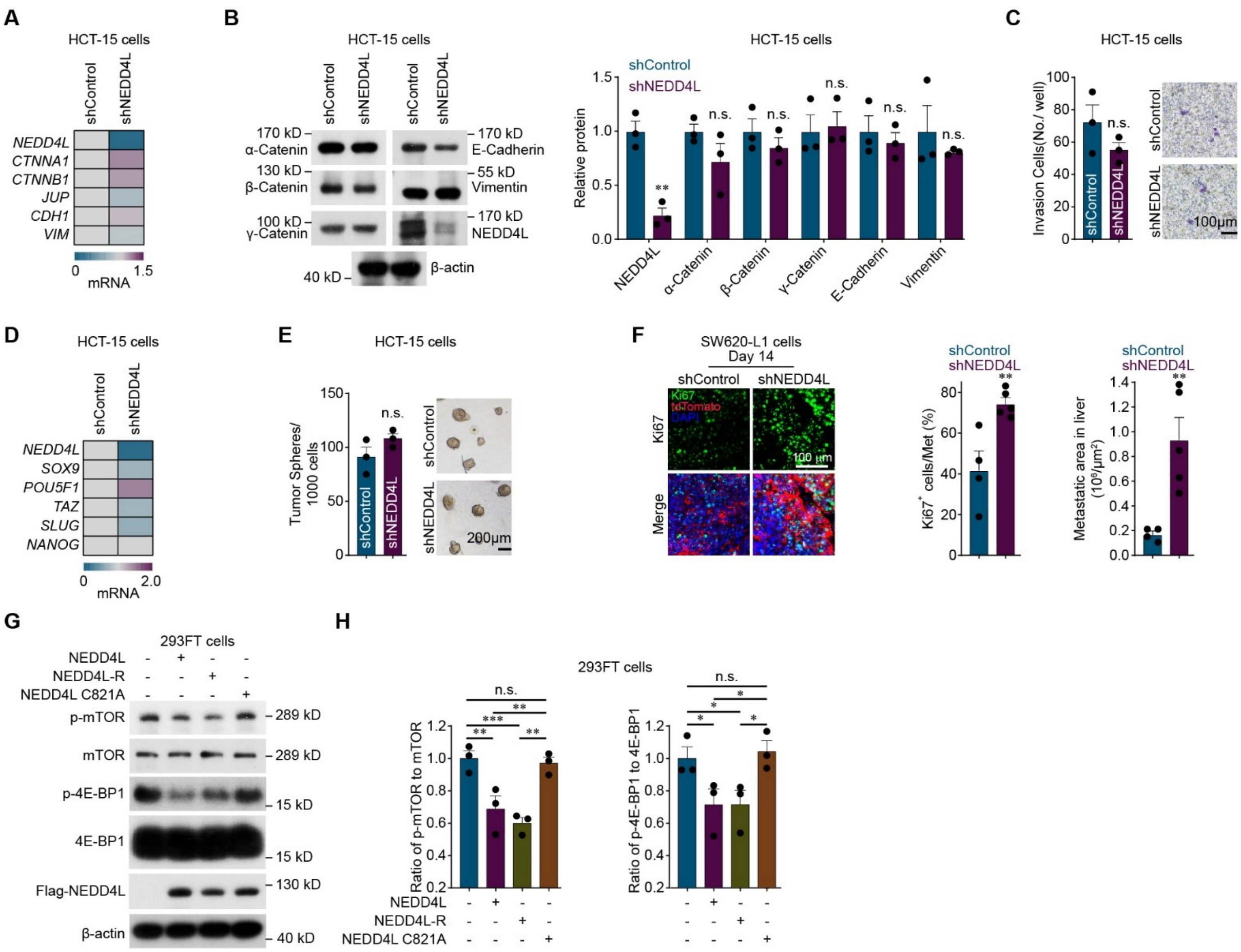
A
HCT-15 cells
shControl
shNEDD4L
NEDD4L
CTNNA1
CTNNB1
JUP
CDH1
VIM
0
1.5
mRNA
B
HCT-15 cells
170 kD
α-Catenin
130 kD
β-Catenin
100 kD
γ-Catenin
40 kD
E-Cadherin
55 kD
Vimentin
NEDD4L
β-actin
Relative protein
n.s.
C
HCT-15 cells
Invasion Cells(No./ well)
100μm
D
SOX9
POU5F1
TAZ
SLUG
NANOG
2.0
E
Tumor Spheres/ 1000 cells
200μm
F
SW620-L1 cells
Day 14
Ki67
tdTomato
DAPI
Merge
100 μm
Ki67+ cells/Met (%)
Metastatic area in liver (10^6/μm^2)
G
293FT cells
NEDD4L-R
NEDD4L C821A
p-mTOR
mTOR
p-4E-BP1
4E-BP1
Flag-NEDD4L
289 kD
15 kD
130 kD
40 kD
H
Ratio of p-mTOR to mTOR
Ratio of p-4E-BP1 to 4E-BP1

Figure S2. NEDD4L decreases colorectal cancer cell proliferation by inhibiting the mTOR signaling pathway.

(A) qPCR analysis of the mRNA expression of EMT markers in shControl or shNEDD4L HCT-15 cells.

(B) Representative western blots (left) and quantification of the protein expression of EMT markers (right) in shControl or shNEDD4L HCT-15 cells.

(C) Quantification (left) and representative images (right) of the Matrigel invasion ability of shControl or shNEDD4L HCT-15 cells (1,000 cells). Scale bar, 100 μm.

(D) qPCR analysis of the mRNA expression of stem cell transcription factors in shControl or shNEDD4L HCT-15 cells.

(E) Quantification (left) and representative images (right) of tumor sphere assay of shControl or shNEDD4L HCT-15 cells (1,000 cells). Scale bar, 200 μm.

(F) Representative images (right), quantification of Ki67 (green)-positive SW620-L1 cells (tdTomato) in the liver metastatic lesions of SW620-L1 cell-implanted BALB/c nude mice (middle), and quantification of metastatic area in liver (right) on day 14 after the intrasplenic injection of shControl or shNEDD4L SW620-L1 cells (1×10^6 cells). Four liver metastatic lesions in 3 mice in the shControl group and 5 liver metastatic lesions in 3 mice in the shNEDD4L group. Scale bar, 100 μm.

(G, H) Representative western blots (G) and quantification of the p-mTOR, mTOR, p-4E-BP1 and 4E-BP1 levels (H) in Control, NEDD4L, NEDD4L-R or NEDD4L C821A 293FT cells.

Three independent experiments were performed (A-E, G and H). The data are presented as the mean ± s.e.m. values. *P*- values were determined by unpaired two-way ANOVA with uncorrected Fisher's LSD test (B), unpaired two-tailed Student's t-test with Welch's correction (C, E and F), or unpaired one-way ANOVA with uncorrected Fisher's LSD test (H). * $P < 0.05$; ** $P < 0.01$; *** $P < 0.001$; n.s., not significant.

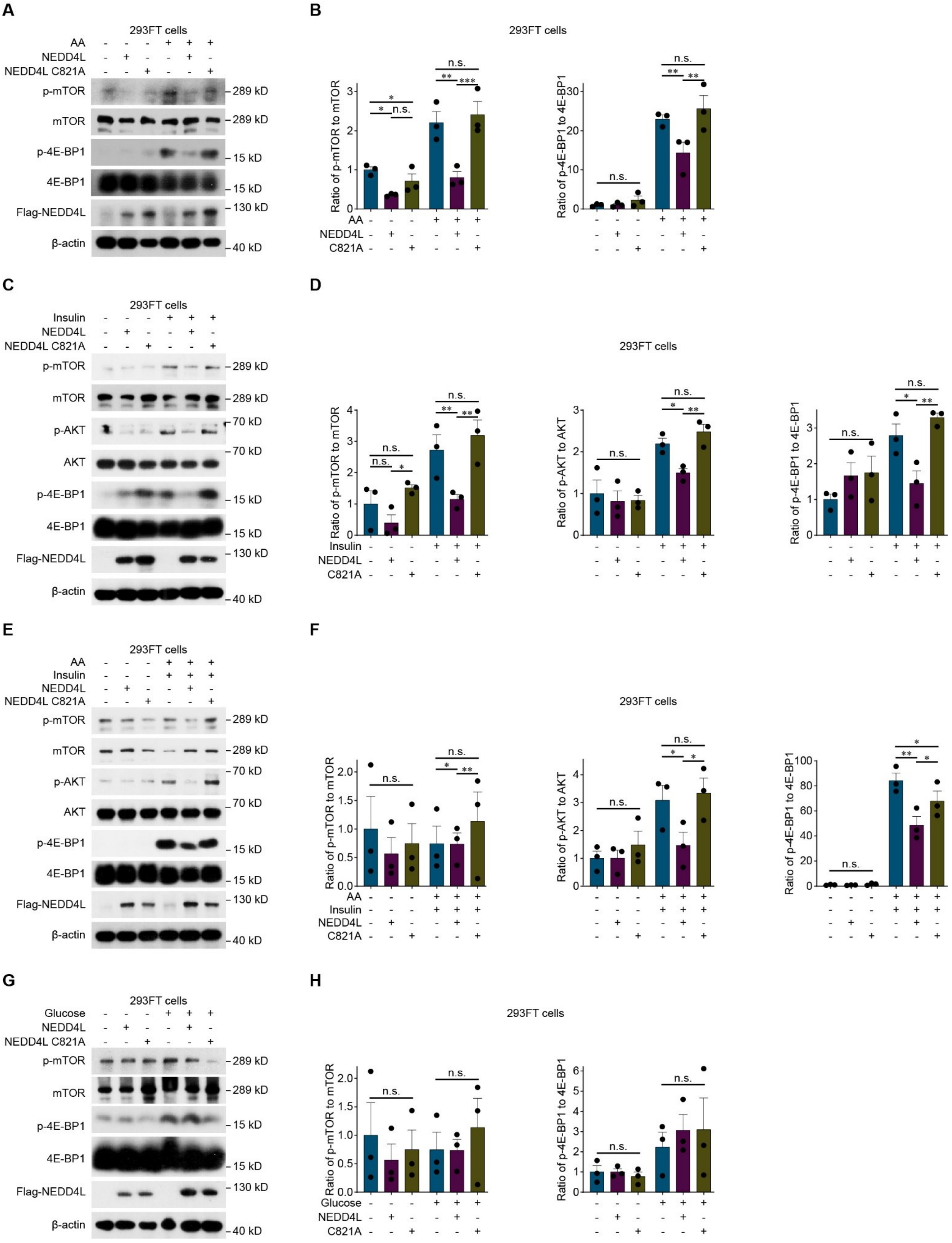

A
293FT cells
AA - - - + + +
NEDD4L - + - - + -
NEDD4L C821A - - + - - +
p-mTOR
mTOR
p-4E-BP1
4E-BP1
Flag-NEDD4L
β-actin
289 kD
289 kD
15 kD
15 kD
130 kD
40 kD
B
293FT cells
Ratio of p-mTOR to mTOR
Ratio of p-4E-BP1 to 4E-BP1
AA
NEDD4L
C821A
n.s.
C
293FT cells
Insulin - - - + + +
NEDD4L - + - - + -
NEDD4L C821A - - + - - +
p-mTOR
mTOR
p-AKT
AKT
p-4E-BP1
4E-BP1
Flag-NEDD4L
β-actin
70 kD
D
293FT cells
Ratio of p-mTOR to mTOR
Ratio of p-AKT to AKT
Ratio of p-4E-BP1 to 4E-BP1
Insulin
NEDD4L
C821A
E
293FT cells
AA - - - + + +
Insulin - - - + + +
NEDD4L - + - - + -
NEDD4L C821A - - + - - +
F
293FT cells
G
293FT cells
Glucose - - - + + +
NEDD4L - + - - + -
NEDD4L C821A - - + - - +
H
293FT cells
Glucose

Figure S3. NEDD4L inhibits the AKT/mTOR signaling pathway under stimulation with AA and insulin.

(A, B) Representative western blots (A) and quantification of the p-mTOR, mTOR, p-4E-BP1 and 4E-BP1 levels (B) in Control, NEDD4L or NEDD4L C821A 293FT cells incubated with or without 200 μM amino acids (AA) for 15 min.

(C, D) Representative western blots (C) and quantification of the p-mTOR, mTOR, p-4E-BP1 and 4E-BP1 levels (D) in Control, NEDD4L or NEDD4L C821A 293FT cells incubated with or without 800 nM insulin for 10 min.

(E, F) Representative western blots (E) and quantification of the p-mTOR, mTOR, p-4E-BP1 and 4E-BP1 levels (F) in Control, NEDD4L or NEDD4L C821A 293FT cells incubated with or without 200 μM AA for 15 min, and 800 nM insulin for 10 min.

(G, H) Representative western blots (G) and quantification of the p-mTOR, mTOR, p-4E-BP1 and 4E-BP1 levels (H) in Control, NEDD4L or NEDD4L C821A 293FT cells incubated with or without 4.5 mg/mL glucose for 20 min.

Three independent experiments were performed (A-H). The data are presented as the mean ± s.e.m. values. *P*- values were determined by unpaired two-way ANOVA with uncorrected Fisher's LSD test (B, D, F, and H). * $P < 0.05$; ** $P < 0.01$; *** $P < 0.001$; n.s., not significant.

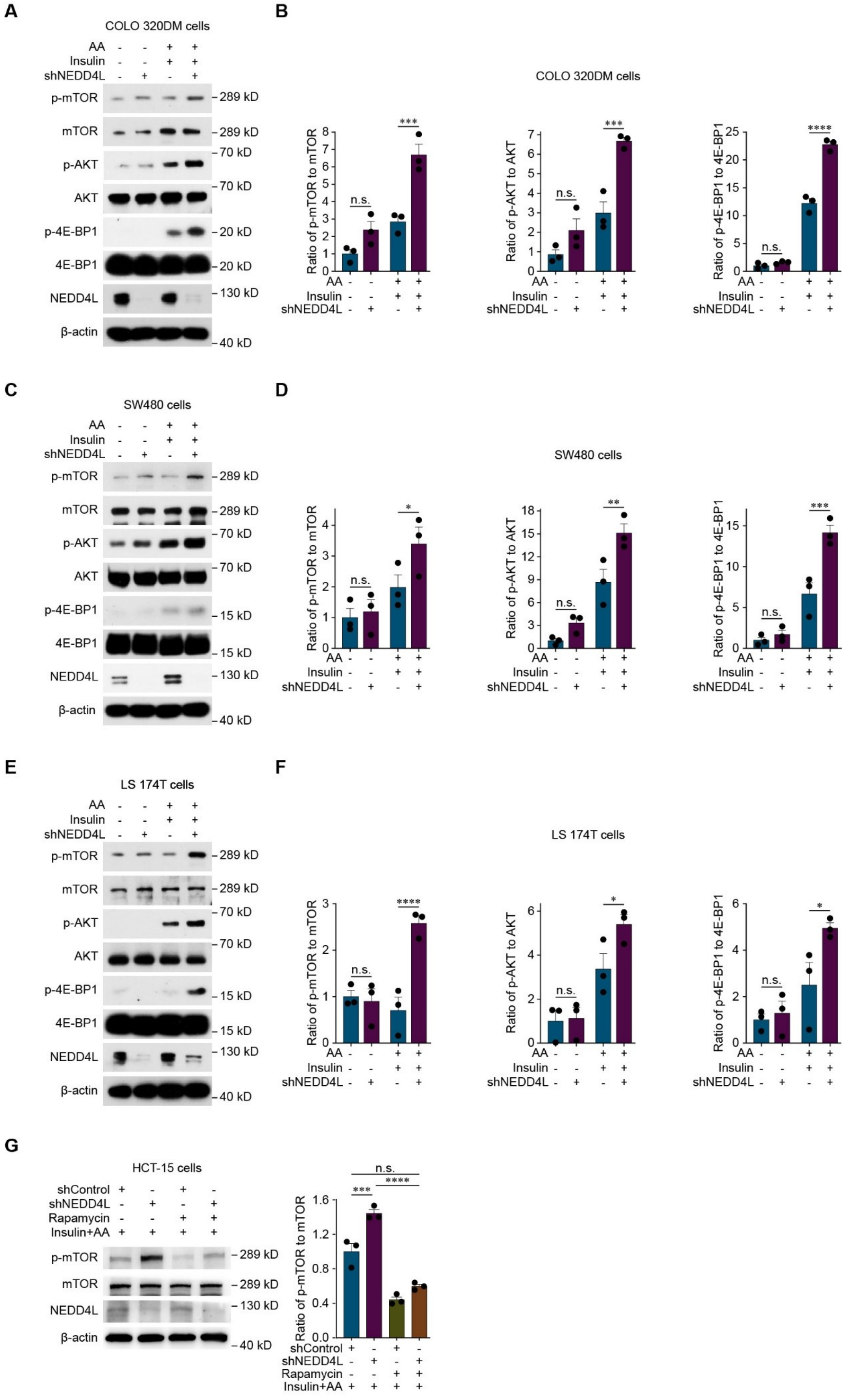
A
COLO 320DM cells
AA - - + +
Insulin - - + +
shNEDD4L - + - +
p-mTOR
mTOR
p-AKT
AKT
p-4E-BP1
4E-BP1
NEDD4L
β-actin
289 kD
289 kD
70 kD
70 kD
20 kD
20 kD
130 kD
40 kD
B
COLO 320DM cells
Ratio of p-mTOR to mTOR
Ratio of p-AKT to AKT
Ratio of p-4E-BP1 to 4E-BP1
n.s.
***
****
C
SW480 cells
AA - - + +
Insulin - - + +
shNEDD4L - + - +
p-mTOR
mTOR
p-AKT
AKT
p-4E-BP1
4E-BP1
NEDD4L
β-actin
289 kD
289 kD
70 kD
70 kD
15 kD
15 kD
130 kD
40 kD
D
SW480 cells
Ratio of p-mTOR to mTOR
Ratio of p-AKT to AKT
Ratio of p-4E-BP1 to 4E-BP1
n.s.
*
**
***
E
LS 174T cells
AA - - + +
Insulin - - + +
shNEDD4L - + - +
p-mTOR
mTOR
p-AKT
AKT
p-4E-BP1
4E-BP1
NEDD4L
β-actin
289 kD
289 kD
70 kD
70 kD
15 kD
15 kD
130 kD
40 kD
F
LS 174T cells
Ratio of p-mTOR to mTOR
Ratio of p-AKT to AKT
Ratio of p-4E-BP1 to 4E-BP1
n.s.
****
*
G
HCT-15 cells
shControl + - + -
shNEDD4L - + - +
Rapamycin - - + +
Insulin+AA + + + +
p-mTOR
mTOR
NEDD4L
β-actin
289 kD
289 kD
130 kD
40 kD
Ratio of p-mTOR to mTOR
n.s.
***
****

Figure S4. Knockdown of NEDD4L in colorectal cancer cells promotes the activation of the AKT/mTOR signaling pathway under stimulation with AA and insulin.

(A, B) Representative western blots (A) and quantification of the p-mTOR, mTOR, p-AKT, AKT, p-4E-BP1 and 4E-BP1 levels (B) in shControl or shNEDD4L COLO 320DM cells incubated with or without 200 μм AA for 15 min and 800 nм insulin for 10 min.

(C, D) Representative western blots (C) and quantification of the p-mTOR, mTOR, p-AKT, AKT, p-4E-BP1 and 4E-BP1 levels (D) in shControl or shNEDD4L SW480 cells incubated with or without 200 μм AA for 15 min and 800 nм insulin for 10 min.

(E, F) Representative western blots (E) and quantification of the p-mTOR, mTOR, p-AKT, AKT, p-4E-BP1 and 4E-BP1 levels (F) in shControl or shNEDD4L LS 174T cells incubated with or without 200 μм AA for 15 min and 800 nм insulin for 10 min.

(G) Representative western blots (left) and quantification of the p-mTOR and mTOR levels (right) in shControl or shNEDD4L HCT-15 cells cultured with or without 100 nм rapamycin for 24 hr in combination with 200 μм AA for 15 min and 800 nм insulin for 10 min.

Three independent experiments were performed (A-G). The data are presented as the mean ± s.e.m. values. *P*- values were determined by unpaired two-way ANOVA with uncorrected Fisher's LSD test (B, D, and F), or unpaired one-way ANOVA with uncorrected Fisher's LSD test (G). * $P < 0.05$; ** $P < 0.01$; *** $P < 0.001$; **** $P < 0.0001$; n.s., not significant.

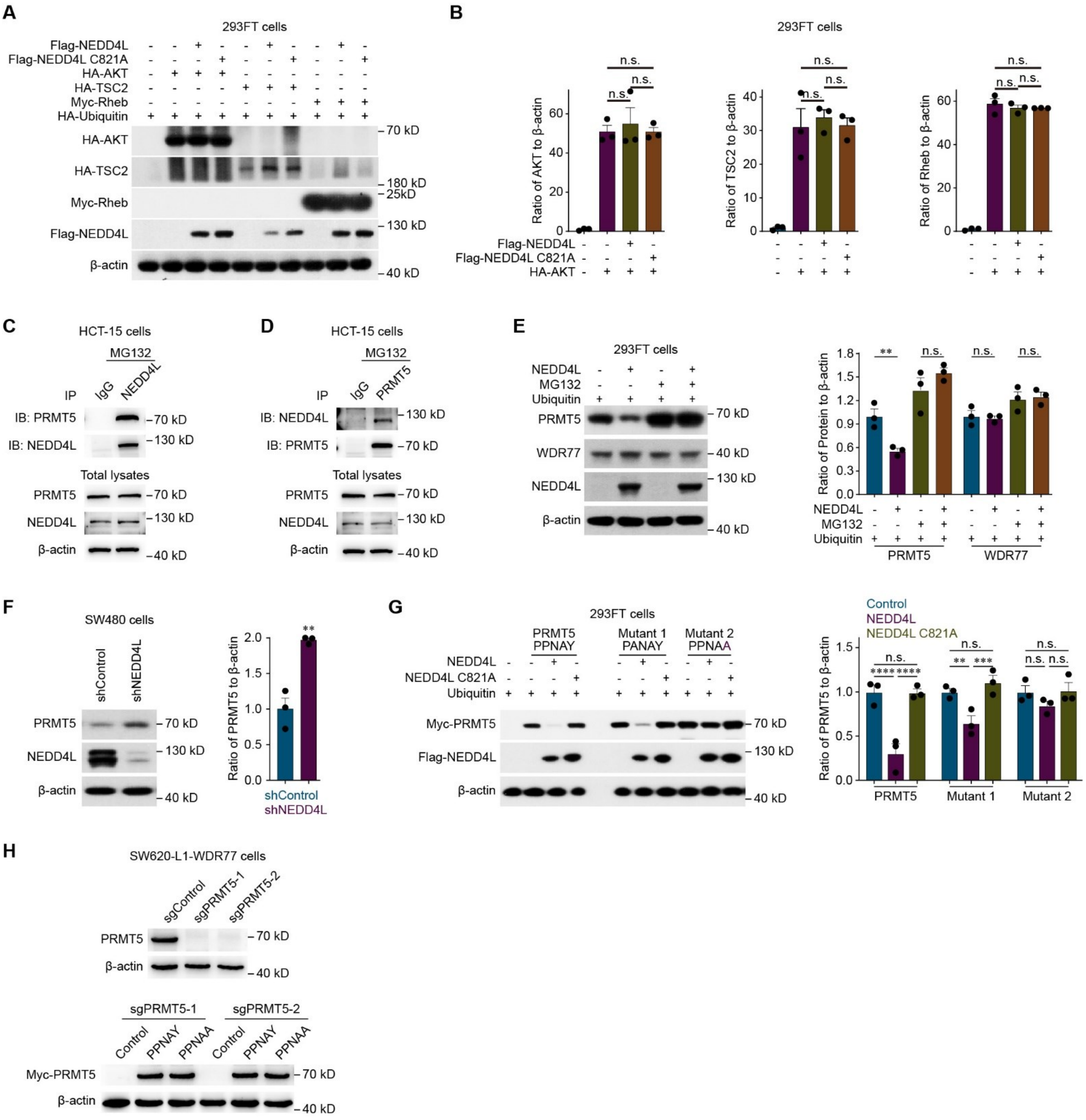

A
293FT cells
Flag-NEDD4L
Flag-NEDD4L C821A
HA-AKT
HA-TSC2
Myc-Rheb
HA-Ubiquitin
HA-AKT
HA-TSC2
Myc-Rheb
Flag-NEDD4L
β-actin
70 kD
180 kD
25kD
130 kD
40 kD
B
293FT cells
Ratio of AKT to β-actin
Ratio of TSC2 to β-actin
Ratio of Rheb to β-actin
n.s.
Flag-NEDD4L
Flag-NEDD4L C821A
HA-AKT
C
HCT-15 cells
MG132
IP
IgG
NEDD4L
IB: PRMT5
IB: NEDD4L
Total lysates
PRMT5
NEDD4L
β-actin
70 kD
130 kD
40 kD
D
HCT-15 cells
MG132
IP
IgG
PRMT5
IB: NEDD4L
IB: PRMT5
Total lysates
PRMT5
NEDD4L
β-actin
E
293FT cells
NEDD4L
MG132
Ubiquitin
PRMT5
WDR77
NEDD4L
β-actin
Ratio of Protein to β-actin
**
n.s.
PRMT5
WDR77
F
SW480 cells
shControl
shNEDD4L
PRMT5
NEDD4L
β-actin
Ratio of PRMT5 to β-actin
**
shControl
shNEDD4L
G
293FT cells
PRMT5
PPNAY
Mutant 1
PANAY
Mutant 2
PPNAA
NEDD4L
NEDD4L C821A
Ubiquitin
Myc-PRMT5
Flag-NEDD4L
β-actin
Control
NEDD4L
NEDD4L C821A
Ratio of PRMT5 to β-actin
n.s.
****
**
***
PRMT5
Mutant 1
Mutant 2
H
SW620-L1-WDR77 cells
sgControl
sgPRMT5-1
sgPRMT5-2
PRMT5
β-actin
70 kD
40 kD
sgPRMT5-1
sgPRMT5-2
Control
PPNAY
PPNAA
Myc-PRMT5
β-actin

Figure S5. NEDD4L ubiquitinates PRMT5 to promote its degradation.

(A, B) Representative western blots (A) and quantification of HA-AKT, HA-TSC2, and Myc-Rheb expression (B) in Control, Flag-NEDD4L or Flag-NEDD4L C821A 293FT cells with HA-ubiquitin overexpression transfected with or without HA-AKT, HA-TSC2, or Myc-Rheb.

(C, D) Co-IPs of endogenous NEDD4L and PRMT5 pulled down by NEDD4L (C) or PRMT5 (D) in HCT-15 cells. The cancer cells were incubated with 20 μм MG132 for 12 hr.

(E) Representative western blots (left) and quantification of endogenous PRMT5 expression and WDR77 expression (right) in Control or NEDD4L 293FT cells overexpressing HA-ubiquitin incubated with or without 20 μм MG132 for 12 hr.

(F) Representative western blots (left) and quantification of PRMT5 expression (right) in shControl or shNEDD4L SW480 cells.

(G) Representative western blots (left) and quantification of the expression of exogenous Flag-PRMT5 or its mutant expression (right) in NEDD4L or NEDD4L C821A 293FT cells in combination with ubiquitin and transfected with PRMT5 containing the wild-type NEDD4L binding motif (PRMT5, PPNAY) or one of two mutant NEDD4L binding motifs (Mutant1, PANAY; Mutant2, PPNAA).

(H) Representative western blots showing PRMT5 expression in sgControl, shPRMT5-1 or sgPRMT5-2 SW620-L1 cells (top), and sgPRMT5-1/Control/WDR77, sgPRMT5-1/PPNAY/WDR77, sgPRMT5-1/PPNAA/WDR77, sgPRMT5-1/Control/WDR77, sgPRMT5-1/PPNAY/WDR77 or sgPRMT5-1/PPNAA/WDR77 SW620-L1 cells. (bottom).

Three independent experiments were performed (A-H). The data are presented as the mean ± s.e.m. values. *P*- values were determined by unpaired one-way ANOVA with uncorrected Fisher's LSD test (B), unpaired two-way ANOVA with uncorrected Fisher's LSD test (E and G), or unpaired two-tailed Student's t-test with Welch's correction (F). ** $P < 0.01$; *** $P < 0.001$; **** $P < 0.0001$; n.s., not significant.

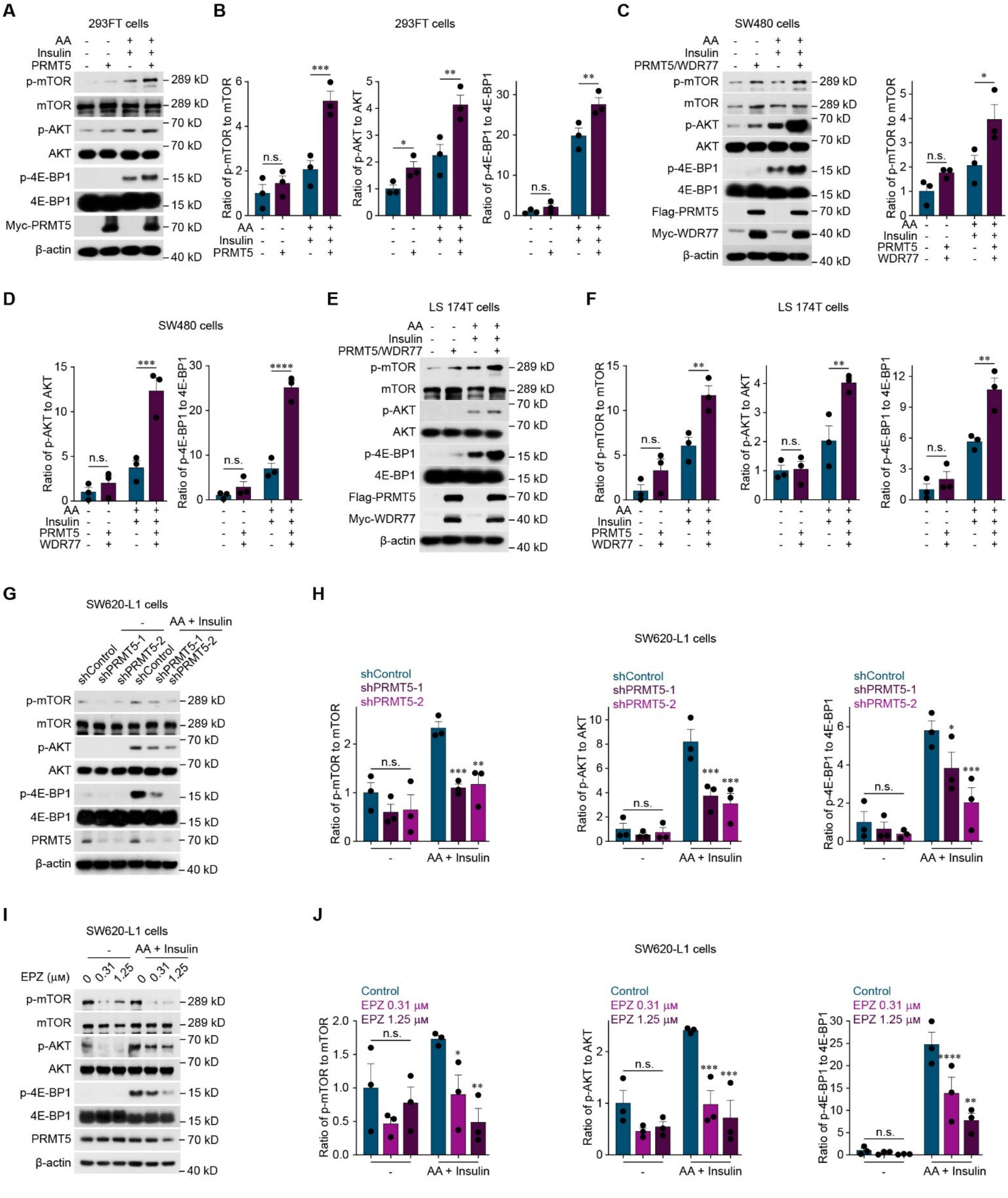

A
293FT cells
AA
Insulin
PRMT5
p-mTOR
mTOR
p-AKT
AKT
p-4E-BP1
4E-BP1
Myc-PRMT5
β-actin
289 kD
70 kD
15 kD
40 kD
B
293FT cells
Ratio of p-mTOR to mTOR
Ratio of p-AKT to AKT
Ratio of p-4E-BP1 to 4E-BP1
n.s.
C
SW480 cells
PRMT5/WDR77
Flag-PRMT5
Myc-WDR77
WDR77
D
SW480 cells
E
LS 174T cells
F
LS 174T cells
G
SW620-L1 cells
AA + Insulin
shControl
shPRMT5-1
shPRMT5-2
PRMT5
H
SW620-L1 cells
I
SW620-L1 cells
EPZ (μM)
0
0.31
1.25
J
SW620-L1 cells
Control
EPZ 0.31 μM
EPZ 1.25 μM

Figure S6. PRMT5 activates the AKT/mTOR signaling pathway.

(A, B) Representative western blots (A) and quantification of the p-mTOR, mTOR, p-AKT, AKT, p-4E-BP1 and 4E-BP1 levels (B) in Control or PRMT5 293FT cells incubated with or without 200 μM AA for 15 min and 800 nM insulin for 10 min.

(C, D) Representative western blots (C, left) and quantification of the p-mTOR, mTOR, p-AKT, AKT, p-4E-BP1 and 4E-BP1 levels (C, right and D) in Control or PRMT5/WDR77 SW480 cells incubated with or without 200 μM AA for 15 min and 800 nM insulin for 10 min.

(E, F) Representative western blots (E) and quantification of the p-mTOR, mTOR, p-AKT, AKT, p-4E-BP1 and 4E-BP1 levels (F) in Control or PRMT5/WDR77 LS 174T cells incubated with or without 200 μM AA for 15 min and 800 nM insulin for 10 min.

(G, H) Representative western blots (G) and quantification of the p-mTOR, mTOR, p-AKT, AKT, p-4E-BP1 and 4E-BP1 levels (H) in shControl, shPRMT5-1 or shPRMT5-2 SW620-L1 cells incubated with or without 200 μM AA for 15 min and 800 nM insulin for 10 min.

(I, J) Representative western blots (I) and quantification of the p-mTOR, mTOR, p-AKT, AKT, p-4E-BP1 and 4E-BP1 levels (J) in SW620-L1 cells treated with the PRMT5 inhibitor EPZ015666 (0, 0.31 or 1.25 μM) for 48 hr and incubated with 200 μM AA for 15 min and 800 nM insulin for 10 min.

Three independent experiments were performed (A-J). The data are presented as the mean ± s.e.m. values. *P*- values were determined by unpaired two-way ANOVA with uncorrected Fisher’s LSD test (B-D, F, H, and J). * $P < 0.05$; ** $P < 0.01$; *** $P < 0.001$; **** $P < 0.0001$; n.s., not significant.

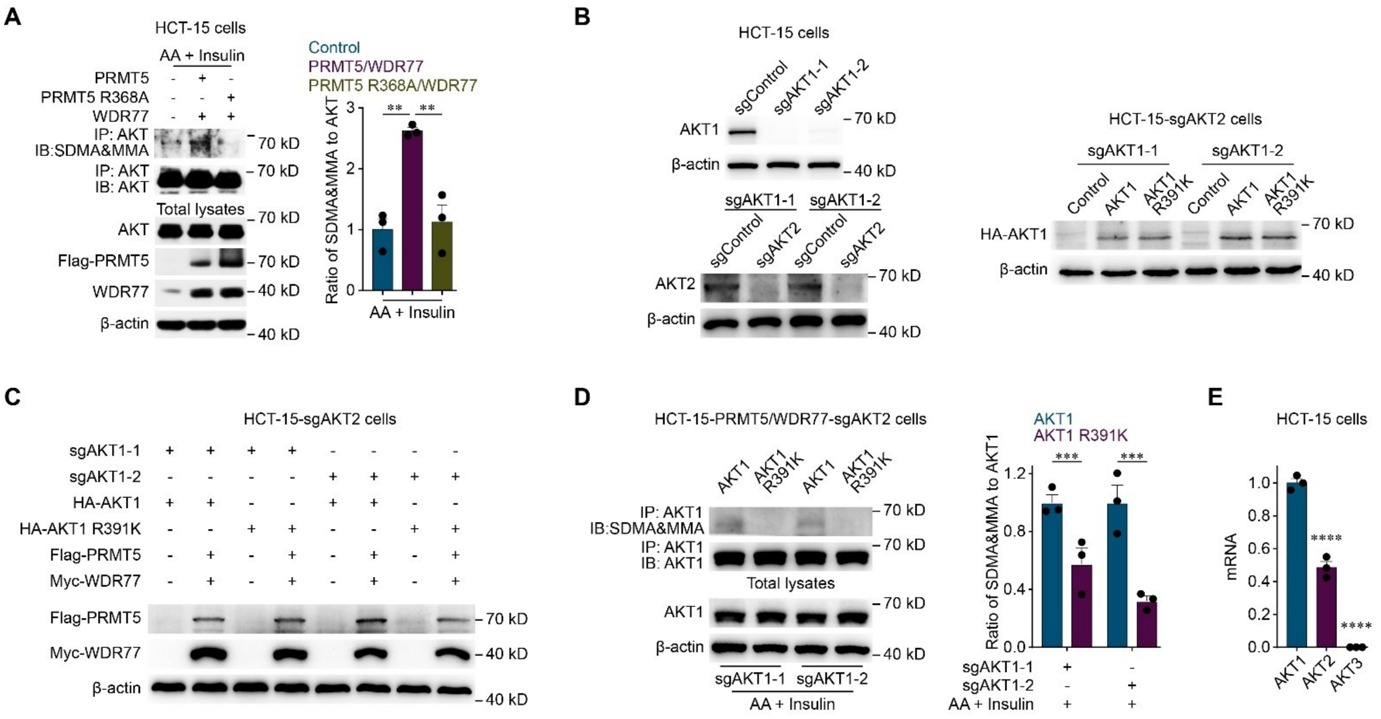

A
HCT-15 cells
AA + Insulin
PRMT5
PRMT5 R368A
WDR77
IP: AKT
IB:SDMA&MMA
IP: AKT
IB: AKT
Total lysates
AKT
Flag-PRMT5
WDR77
β-actin
70 kD
40 kD
Control
PRMT5/WDR77
PRMT5 R368A/WDR77
Ratio of SDMA&MMA to AKT
**
AA + Insulin
B
HCT-15 cells
sgControl
sgAKT1-1
sgAKT1-2
AKT1
β-actin
sgAKT2
AKT2
HCT-15-sgAKT2 cells
Control
AKT1
AKT1 R391K
HA-AKT1
C
HCT-15-sgAKT2 cells
HA-AKT1 R391K
Myc-WDR77
D
HCT-15-PRMT5/WDR77-sgAKT2 cells
IP: AKT1
IB:SDMA&MMA
IB: AKT1
AKT1 R391K
Ratio of SDMA&MMA to AKT1
***
E
mRNA
****
AKT2
AKT3

Figure S7. PRMT5 methylates R391 in AKT1.

(A) Representative western blots (left) and quantification of pan-AKT methylarginine and AKT levels (right) in Control, PRMT5/WDR77 or PRMT5 R368A/WDR77 HCT-15 cells incubated with 200 μM AA for 15 min and 800 nM insulin for 10 min.

(B) Representative western blots showing AKT1 expression in sgControl, sgAKT1-1 or sgAKT1-2 HCT-15 cells (top left), AKT2 expression in sgControl/sgControl, sgAKT1-1/sgAKT2 or sgAKT1-2/sgAKT2 HCT-15 cells (bottom left), and HA-AKT1 and HA-AKT R391K expression in sgAKT1-1/sgAKT2 or sgAKT1-2/sgAKT2 HCT-15 cells with wild-type AKT1 or the AKT1 R391K mutant that cannot be methylated by PRMT5 (right).

(C) Representative western blots showing Flag-PRMT5 and Myc-WDR77 expression in HCT-15 cells with Control or PRMT5 and WDR77 overexpression in combination with endogenous AKT1 and AKT2 knockout and restoration with wild-type AKT1, or the AKT1-R391K mutant that cannot be methylated by PRMT5 (right).

(D) Representative western blots (left) and quantification of AKT1 methylarginine levels and AKT1 expression (right) in HCT-15 cells with PRMT5 and WDR77 overexpression in combination with endogenous AKT1 and AKT2 knockout and restoration with wild-type AKT1, or the AKT1-R391K mutant that cannot be methylated by PRMT5 incubated with 200 μM AA for 15 min and 800 nM insulin for 10 min.

(E) qPCR analysis of the mRNA expression of AKT1, AKT2 and AKT3 in HCT-15 cells.

Three independent experiments were performed (A-E). The data are presented as the mean ± s.e.m. values. *P*- values were determined by unpaired one-way ANOVA with uncorrected Fisher's LSD test (A, E), or unpaired two-way ANOVA with uncorrected Fisher's LSD test (D). ** $P < 0.01$; ***, $P < 0.001$; **** $P < 0.0001$; n.s., not significant.

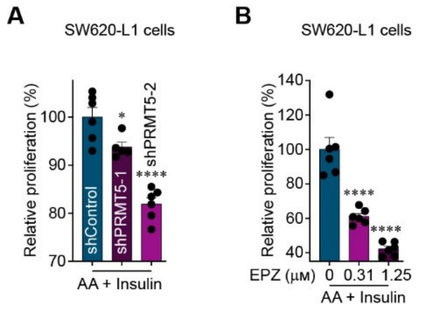

Figure S8. PRMT5 increase colorectal cancer cell proliferation.

(A) *In vitro* proliferation assay of shControl, shPRMT5-1 or shPRMT5-2 SW620-L1 cells (3,000 cells) cultured with 200 μM AA and 800 nM insulin for 24 hr.

(B) *In vitro* proliferation assay of SW620-L1 cells cultured with the PRMT5 inhibitor EPZ015666 (0, 0.31 or 1.25 μM), 200 μM AA acid and 800 nM insulin for 24 hr.

At least five independent experiments were performed (A, B). The data are presented as the mean ± s.e.m. values. *P*- values were determined by unpaired one-way ANOVA with uncorrected Fisher's LSD test (A, B). * $P < 0.05$; **** $P < 0.0001$.

Table S1. The list of shRNAs of the shRNA library targeting 156 human E3 ubiquitin ligases.

Gene ID	NM ID	Gene	shRNA ID
267	NM_001144	AMFR	TRCN0000003373<br>TRCN0000003374<br>TRCN0000003375
10393	NM_014885	ANAPC10	TRCN0000004431<br>TRCN0000004432<br>TRCN0000004433<br>TRCN0000004434<br>TRCN0000010876
51529	NM_016476	ANAPC11	TRCN0000038799<br>TRCN0000038800<br>TRCN0000038801<br>TRCN0000038802<br>TRCN0000038803
29882	NM_013366	ANAPC2	TRCN0000004357<br>TRCN0000004358<br>TRCN0000004359<br>TRCN0000004360<br>TRCN0000010870
29945	NM_013367	ANAPC4	TRCN0000004361<br>TRCN0000004362<br>TRCN0000004363<br>TRCN0000004364<br>TRCN0000004365
51433	NM_016237	ANAPC5	TRCN0000004152<br>TRCN0000004153<br>TRCN0000004154

51433	NM_016237	ANAPC5	TRCN0000004155<br>TRCN0000004156
25820	NM_005744	ARIH1	TRCN0000007500<br>TRCN0000007501<br>TRCN0000007502<br>TRCN0000007503<br>TRCN0000007504
580	NM_000465	BARD1	TRCN0000003743<br>TRCN0000003744<br>TRCN0000003745<br>TRCN0000003746<br>TRCN0000003747
329	NM_001166	BIRC2	TRCN0000003780<br>TRCN0000003781<br>TRCN0000003782<br>TRCN0000003783<br>TRCN0000003784
330	NM_001165	BIRC3	TRCN0000003775<br>TRCN0000003776<br>TRCN0000003777<br>TRCN0000003778<br>TRCN0000003779
648	NM_005180	BMI1	TRCN0000020154<br>TRCN0000020155<br>TRCN0000020156<br>TRCN0000020157<br>TRCN0000020158
8315	NM_006768	BRAP	TRCN0000007629<br>TRCN0000007630<br>TRCN0000007631

8315	NM_006768	BRAP	TRCN0000007632<br>TRCN0000007633
672	NM_007294	BRCA1	TRCN0000009823<br>TRCN0000009824<br>TRCN0000010305<br>TRCN0000039833<br>TRCN0000039834<br>TRCN0000039835<br>TRCN0000039836<br>TRCN0000039837
8945	NM_003939	BTRC	TRCN0000006541<br>TRCN0000006542<br>TRCN0000006543<br>TRCN0000006544<br>TRCN0000006545
283450	NM_173813	C12orf51	TRCN0000004992<br>TRCN0000004993<br>TRCN0000004994<br>TRCN0000004995<br>TRCN0000004996
79596	NM_024546	C13orf7	TRCN0000133667<br>TRCN0000134953<br>TRCN0000136897<br>TRCN0000137082<br>TRCN0000137111
55148	NM_018108	C14orf130	TRCN0000037024<br>TRCN0000037025<br>TRCN0000037026<br>TRCN0000037027<br>TRCN0000037028

79594	NM_024544	C1orf166	TRCN0000033934
			TRCN0000033936
			TRCN0000033937
			TRCN0000033938
55832	NM_018448	CAND1	TRCN0000003459
			TRCN0000003460
			TRCN0000003461
			TRCN0000003462
			TRCN0000003463
867	NM_005188	CBL	TRCN0000010310
			TRCN0000010311
			TRCN0000039723
			TRCN0000039724
			TRCN0000039725
			TRCN0000039726
			TRCN0000039727
57332	NM_020649	CBX8	TRCN0000021894
			TRCN0000021895
			TRCN0000021896
			TRCN0000021897
			TRCN0000021898
8697	NM_004661	CDC23	TRCN0000007375
			TRCN0000007376
			TRCN0000007377
			TRCN0000007378
			TRCN0000007379
55743	NM_018223	CHFR	TRCN0000007702
			TRCN0000007703
			TRCN0000007704
			TRCN0000007705

55743	NM_018223	CHFR	TRCN0000007706
4850	NM_013316	CNOT4	TRCN0000015213<br>TRCN0000015214<br>TRCN0000015215<br>TRCN0000015216<br>TRCN0000015217
8454	NM_003592	CUL1	TRCN0000003391<br>TRCN0000003392<br>TRCN0000003393<br>TRCN0000003394<br>TRCN0000010781
8453	NM_003591	CUL2	TRCN0000006522<br>TRCN0000006523<br>TRCN0000006524<br>TRCN0000006525<br>TRCN0000006526
8452	NM_003590	CUL3	TRCN0000073343<br>TRCN0000073344<br>TRCN0000073345<br>TRCN0000073346<br>TRCN0000073347
8451	NM_003589	CUL4A	TRCN0000006527<br>TRCN0000006528<br>TRCN0000006529<br>TRCN0000006530<br>TRCN0000006531
8450	NM_003588	CUL4B	TRCN0000006532<br>TRCN0000006533<br>TRCN0000006534<br>TRCN0000006535

8450	NM_003588	CUL4B	TRCN0000006536
8065	NM_003478	CUL5	TRCN0000006537<br>TRCN0000006538<br>TRCN0000006539<br>TRCN0000006540<br>TRCN0000011030
9820	NM_014780	CUL7	TRCN0000006480<br>TRCN0000006481<br>TRCN0000006482<br>TRCN0000006483<br>TRCN0000006484
1643	NM_000107	DDB2	TRCN0000083993<br>TRCN0000083994<br>TRCN0000083995<br>TRCN0000083996<br>TRCN0000083997
113878	NM_020892	DTX2	TRCN0000004558<br>TRCN0000004559<br>TRCN0000004560<br>TRCN0000004561
9666	NM_014648	DZIP3	TRCN0000034244<br>TRCN0000034246<br>TRCN0000034247<br>TRCN0000034248
51366	NM_015902	EDD1	TRCN0000003408<br>TRCN0000003409<br>TRCN0000003410<br>TRCN0000003411<br>TRCN0000003412
2033	NM_001429	EP300	TRCN0000009882

2033	NM_001429	EP300	TRCN0000009883<br>TRCN0000009884<br>TRCN0000039883<br>TRCN0000039884<br>TRCN0000039885<br>TRCN0000039886<br>TRCN0000039887
1161	NM_000082	ERCC8	TRCN0000003719<br>TRCN0000003720<br>TRCN0000003721<br>TRCN0000003722<br>TRCN0000003723
55120	NM_018062	FANCL	TRCN0000083298<br>TRCN0000083299<br>TRCN0000083300<br>TRCN0000083301<br>TRCN0000083302
25827	NM_012157	FBXL2	TRCN0000004276<br>TRCN0000004277<br>TRCN0000004278<br>TRCN0000004279<br>TRCN0000004280
26223	NM_012159	FBXL21	TRCN0000004286<br>TRCN0000004287<br>TRCN0000004289<br>TRCN0000010866
26224	NM_012158	FBXL3	TRCN0000004281<br>TRCN0000004282<br>TRCN0000004283<br>TRCN0000004284

26224	NM_012158	FBXL3	TRCN0000004285
26234	NM_012161	FBXL5	TRCN0000004290<br>TRCN0000004291<br>TRCN0000004292<br>TRCN0000004293<br>TRCN0000004294
26233	NM_012162	FBXL6	TRCN0000004295<br>TRCN0000004296<br>TRCN0000004297<br>TRCN0000004298<br>TRCN0000004299
23194	NM_012304	FBXL7	TRCN0000118322<br>TRCN0000118323<br>TRCN0000118324<br>TRCN0000118325<br>TRCN0000118326
80204	NM_012167	FBXO11	TRCN0000004300<br>TRCN0000004301<br>TRCN0000004302<br>TRCN0000004303<br>TRCN0000004304
26232	NM_012168	FBXO2	TRCN0000004305<br>TRCN0000004306<br>TRCN0000004307<br>TRCN0000010867<br>TRCN0000010868
23014	NM_015002	FBXO21	TRCN0000034284<br>TRCN0000034285<br>TRCN0000034286<br>TRCN0000034287

23014	NM_015002	FBXO21	TRCN0000034288
26263	NM_012170	FBXO22	TRCN0000004308<br>TRCN0000004309<br>TRCN0000004310<br>TRCN0000004311<br>TRCN0000010869
26261	NM_012172	FBXO24	TRCN0000004312<br>TRCN0000004313<br>TRCN0000004314<br>TRCN0000004315<br>TRCN0000004316
26260	NM_012173	FBXO25	TRCN0000004317<br>TRCN0000004318<br>TRCN0000004319<br>TRCN0000004320<br>TRCN0000004321
26273	NM_012175	FBXO3	TRCN0000004327<br>TRCN0000004328<br>TRCN0000004329<br>TRCN0000004330<br>TRCN0000004331
26272	NM_012176	FBXO4	TRCN0000034319<br>TRCN0000034320<br>TRCN0000034321<br>TRCN0000034322<br>TRCN0000034323
26270	NM_018438	FBXO6	TRCN0000007731<br>TRCN0000007732<br>TRCN0000007733<br>TRCN0000007734

26270	NM_018438	FBXO6	TRCN0000011101
			TRCN0000004337
			TRCN0000004338
25793	NM_012179	FBXO7	TRCN0000004339
			TRCN0000004340
			TRCN0000004341
			TRCN0000034309
			TRCN0000034310
26268	NM_012347	FBXO9	TRCN0000034311
			TRCN0000034312
			TRCN0000034313
			TRCN0000004342
			TRCN0000004343
23291	NM_012300	FBXW11	TRCN0000004344
			TRCN0000004345
			TRCN0000004346
			TRCN0000006546
			TRCN0000006547
26190	NM_012164	FBXW2	TRCN0000006548
			TRCN0000006549
			TRCN0000006550
			TRCN0000060758
			TRCN0000060759
55527	NM_018708	FEM1A	TRCN0000060760
			TRCN0000060761
			TRCN0000060579
			TRCN0000060580
10116	NM_015322	FEM1B	TRCN0000060581
			TRCN0000060582
57531	NM_020771	HACE1	TRCN0000003413

57531	NM_020771	HACE1	TRCN0000003414<br>TRCN0000003415<br>TRCN0000003416<br>TRCN0000003417
25831	NM_015382	HECTD1	TRCN0000004083<br>TRCN0000004084<br>TRCN0000004085<br>TRCN0000004086<br>TRCN0000004087
143279	NM_173497	HECTD2	TRCN0000007758<br>TRCN0000007759<br>TRCN0000007760<br>TRCN0000007761<br>TRCN0000007762
79654	XM_371246	HECTD3	TRCN0000118242<br>TRCN0000118243<br>TRCN0000118244<br>TRCN0000118245<br>TRCN0000118246
23072	NM_015052	HECW1	TRCN0000001523<br>TRCN0000001524<br>TRCN0000010637<br>TRCN0000010638<br>TRCN0000010639
57520	XM_038999	HECW2	TRCN0000004789<br>TRCN0000004790<br>TRCN0000004791<br>TRCN0000004792<br>TRCN0000004793
8925	NM_003922	HERC1	TRCN0000007243

8925	NM_003922	HERC1	TRCN0000007244
			TRCN0000007245
			TRCN0000007246
			TRCN0000007247
8924	NM_004667	HERC2	TRCN0000007380
			TRCN0000007381
			TRCN0000007382
			TRCN0000007383
			TRCN0000007384
8916	NM_014606	HERC3	TRCN0000000291
			TRCN0000000292
			TRCN0000000293
			TRCN0000000294
			TRCN0000000295
26091	NM_015601	HERC4	TRCN0000034299
			TRCN0000034300
			TRCN0000034301
			TRCN0000034302
			TRCN0000034303
51191	NM_016323	HERC5	TRCN0000004168
			TRCN0000004169
			TRCN0000004170
			TRCN0000004171
			TRCN0000010859
55008	NM_017912	HERC6	TRCN0000158995
			TRCN0000160017
			TRCN0000160044
			TRCN0000160299
10075	NM_031407	HUWE1	TRCN0000073303
			TRCN0000073304

10075	NM_031407	HUWE1	TRCN0000073305<br>TRCN0000073306<br>TRCN0000073307
154214	NM_152553	IBRDC1	TRCN0000034124<br>TRCN0000034125<br>TRCN0000034126<br>TRCN0000034127<br>TRCN0000034128
255488	NM_182757	IBRDC2	TRCN0000034149<br>TRCN0000034150<br>TRCN0000034151<br>TRCN0000034152<br>TRCN0000034153
83737	NM_031483	ITCH	TRCN0000002087<br>TRCN0000002088<br>TRCN0000002089<br>TRCN0000002090<br>TRCN0000010680
9870	NM_014821	KIAA0317	TRCN0000004423<br>TRCN0000004424<br>TRCN0000004425<br>TRCN0000004426<br>TRCN0000010874
55632	NM_017769	KIAA1333	TRCN0000004220<br>TRCN0000004221<br>TRCN0000004222<br>TRCN0000004223<br>TRCN0000004224
4008	NM_005358	LMO7	TRCN0000006489<br>TRCN0000006490

4008	NM_005358	LMO7	TRCN0000006491<br>TRCN0000006492<br>TRCN0000006493
222484	NM_153371	LNX2	TRCN0000007745<br>TRCN0000007746<br>TRCN0000007747<br>TRCN0000007748<br>TRCN0000011197
164832	NM_198461	LONRF2	TRCN0000007825<br>TRCN0000007826<br>TRCN0000007827<br>TRCN0000007828<br>TRCN0000007829
79836	NM_024778	LONRF3	TRCN0000022424<br>TRCN0000022425<br>TRCN0000022426<br>TRCN0000022427<br>TRCN0000022428
10892	NM_006785	MALT1	TRCN0000073823<br>TRCN0000073824<br>TRCN0000073825<br>TRCN0000073826<br>TRCN0000222552
64844	NM_022826	MARCH7	TRCN0000073068<br>TRCN0000073070<br>TRCN0000073072<br>TRCN0000222575<br>TRCN0000222576
57574	NM_020814	MARCHF4	TRCN0000127530<br>TRCN0000129384

57574	NM_020814	MARCHF4	TRCN0000130947<br>TRCN0000130948<br>TRCN0000130962
54708	NM_017824	MARCHF5	TRCN0000037014<br>TRCN0000037015<br>TRCN0000037016<br>TRCN0000037017<br>TRCN0000037018
92979	NM_138396	MARCHF9	TRCN0000073168<br>TRCN0000073169<br>TRCN0000073170<br>TRCN0000073171<br>TRCN0000073172
4193	NM_002392	MDM2	TRCN0000003376<br>TRCN0000003377<br>TRCN0000003378<br>TRCN0000003379<br>TRCN0000003380
112950	NM_001001651	MED8	TRCN0000052948<br>TRCN0000052949<br>TRCN0000052950<br>TRCN0000052951<br>TRCN0000052952<br>TRCN0000174212
23295	NM_015246	MGRN1	TRCN0000033789<br>TRCN0000033790<br>TRCN0000033791<br>TRCN0000033792<br>TRCN0000033793
54542	NM_018835	MNAB	TRCN0000037009

54542	NM_018835	MNAB	TRCN0000037012<br>TRCN0000037013
29116	NM_013262	MYLIP	TRCN0000033819<br>TRCN0000033820<br>TRCN0000033821<br>TRCN0000033822<br>TRCN0000033823
4734	NM_006154	NEDD4	TRCN0000007550<br>TRCN0000007551<br>TRCN0000007552<br>TRCN0000007553<br>TRCN0000007554
23327	NM_015277	NEDD4L	TRCN0000000904<br>TRCN0000000905<br>TRCN0000000906<br>TRCN0000000907<br>TRCN0000000908
51070	NM_015953	NOSIP	TRCN0000045603<br>TRCN0000045604<br>TRCN0000045605<br>TRCN0000045606<br>TRCN0000045607
5071	NM_013988	PARK2	TRCN0000000281<br>TRCN0000000282<br>TRCN0000000283<br>TRCN0000000284<br>TRCN0000000285
84108	NM_032154	PCGF6	TRCN0000073108<br>TRCN0000073109<br>TRCN0000073110

84108	NM_032154	PCGF6	TRCN0000073111<br>TRCN0000073112
23759	NM_014337	PPIL2	TRCN0000000160<br>TRCN0000000161<br>TRCN0000000162<br>TRCN0000000163<br>TRCN0000000164
27339	NM_014502	PRPF19	TRCN0000006592<br>TRCN0000006593<br>TRCN0000006594<br>TRCN0000006595<br>TRCN0000006596
5930	NM_006910	RBBP6	TRCN0000034214<br>TRCN0000034215<br>TRCN0000034216<br>TRCN0000034217<br>TRCN0000034218
149041	NM_172071	RC3H1	TRCN0000122428<br>TRCN0000122593<br>TRCN0000122891<br>TRCN0000139015<br>TRCN0000139513<br>TRCN0000139559<br>TRCN0000140092<br>TRCN0000142153
149041	NM_172071	RC3H1	TRCN0000142634<br>TRCN0000144045
6015	NM_002931	RING1	TRCN0000021989<br>TRCN0000021990<br>TRCN0000021991

6015	NM_002931	RING1	TRCN0000021992<br>TRCN0000021993
26994	NM_014372	RNF11	TRCN0000038794<br>TRCN0000038795<br>TRCN0000038796<br>TRCN0000038797<br>TRCN0000038798
51132	NM_016120	RNF12	TRCN0000004139<br>TRCN0000004140<br>TRCN0000004141<br>TRCN0000004142<br>TRCN0000004143
55298	NM_018320	RNF121	TRCN0000007721<br>TRCN0000007722<br>TRCN0000007723<br>TRCN0000007724<br>TRCN0000007725
54941	NM_017831	RNF125	TRCN0000004230<br>TRCN0000004231<br>TRCN0000004232<br>TRCN0000004233<br>TRCN0000004234
79589	NM_024539	RNF128	TRCN0000004794<br>TRCN0000004795<br>TRCN0000004796<br>TRCN0000004797<br>TRCN0000004798
55819	NM_018434	RNF130	TRCN0000007726<br>TRCN0000007727<br>TRCN0000007728

55819	NM_018434	RNF130	TRCN0000007729
			TRCN0000007730
168433	NM_139175	RNF133	TRCN0000011147
			TRCN0000011148
			TRCN0000011149
			TRCN0000011150
			TRCN0000011151
9604	NM_004290	RNF14	TRCN0000003442
			TRCN0000003443
			TRCN0000003444
			TRCN0000010785
			TRCN0000010786
378925	NM_198085	RNF148	TRCN0000004799
			TRCN0000004800
			TRCN0000004801
			TRCN0000004802
			TRCN0000004803
284996	NM_173647	RNF149	TRCN0000034154
			TRCN0000034155
			TRCN0000034156
			TRCN0000034157
			TRCN0000034158
57484	NM_020724	RNF150	TRCN0000118552
			TRCN0000118553
			TRCN0000118554
			TRCN0000118555
			TRCN0000118556
220441	NM_173557	RNF152	TRCN0000007763
			TRCN0000007764
			TRCN0000007765

220441	NM_173557	RNF152	TRCN0000007766<br>TRCN0000007767
26001	NM_015528	RNF167	TRCN0000004097<br>TRCN0000004098<br>TRCN0000004099<br>TRCN0000004100<br>TRCN0000004101
285533	NM_173662	RNF175	TRCN0000007768<br>TRCN0000007769<br>TRCN0000007770<br>TRCN0000007771<br>TRCN0000007772
54546	NM_019062	RNF186	TRCN0000004502<br>TRCN0000004503<br>TRCN0000004504<br>TRCN0000004505<br>TRCN0000004506
25897	NM_015435	RNF19	TRCN0000004804<br>TRCN0000004805<br>TRCN0000004806<br>TRCN0000004807<br>TRCN0000004808
162333	NM_152598	RNF190	TRCN0000073213<br>TRCN0000073214<br>TRCN0000073215<br>TRCN0000073216<br>TRCN0000073217
6045	NM_007212	RNF2	TRCN0000033694<br>TRCN0000033695<br>TRCN0000033696

6045	NM_007212	RNF2	TRCN0000033697<br>TRCN0000033698
56254	NM_019592	RNF20	TRCN0000033874<br>TRCN0000033875<br>TRCN0000033876<br>TRCN0000033877<br>TRCN0000033878
9810	NM_014771	RNF40	TRCN0000004780<br>TRCN0000004781<br>TRCN0000004782<br>TRCN0000004783<br>TRCN0000004784
9616	NM_014245	RNF7	TRCN0000038804<br>TRCN0000038805<br>TRCN0000038806<br>TRCN0000038807<br>TRCN0000038808
9025	NM_003958	RNF8	TRCN0000003437<br>TRCN0000003438<br>TRCN0000003439<br>TRCN0000003440<br>TRCN0000003441
6502	NM_005983	SKP2	TRCN0000007530<br>TRCN0000007531<br>TRCN0000007532<br>TRCN0000007533<br>TRCN0000007534
57154	NM_020429	SMURF1	TRCN0000003471<br>TRCN0000003472<br>TRCN0000003473

57154	NM_020429	SMURF1	TRCN0000003474<br>TRCN0000010791
64750	NM_022739	SMURF2	TRCN0000003475<br>TRCN0000003476<br>TRCN0000003477<br>TRCN0000003478<br>TRCN0000010792
10273	NM_005861	STUB1	TRCN0000007525<br>TRCN0000007526<br>TRCN0000007527<br>TRCN0000007528<br>TRCN0000007529
6921	NM_005648	TCEB1	TRCN0000022124<br>TRCN0000022125<br>TRCN0000022126<br>TRCN0000022127<br>TRCN0000022128
6923	NM_007108	TCEB2	TRCN0000007661<br>TRCN0000007662<br>TRCN0000011096<br>TRCN0000011097<br>TRCN0000011098
7188	NM_004619	TRAF5	TRCN0000007343<br>TRCN0000007344<br>TRCN0000007345<br>TRCN0000007346<br>TRCN0000007347
7189	NM_004620	TRAF6	TRCN0000007348<br>TRCN0000007349<br>TRCN0000007350

7189	NM_004620	TRAF6	TRCN0000007351<br>TRCN0000007352
84231	NM_032271	TRAF7	TRCN0000056948<br>TRCN0000056949<br>TRCN0000056950<br>TRCN0000056951<br>TRCN0000056952
54476	NM_019011	TRIAD3	TRCN0000003464<br>TRCN0000003465<br>TRCN0000003466<br>TRCN0000010788<br>TRCN0000010789
373	NM_001656	TRIM23	TRCN0000034204<br>TRCN0000034205<br>TRCN0000034206<br>TRCN0000034208
22954	NM_012210	TRIM32	TRCN0000003455<br>TRCN0000003456<br>TRCN0000003457<br>TRCN0000003458<br>TRCN0000010787
79097	NM_024114	TRIM48	TRCN0000033919<br>TRCN0000033920<br>TRCN0000033921<br>TRCN0000033922<br>TRCN0000033923
57093	NM_020358	TRIM49	TRCN0000033884<br>TRCN0000033885<br>TRCN0000033886<br>TRCN0000033887

57093	NM_020358	TRIM49	TRCN0000033888
84676	NM_032588	TRIM63	TRCN0000073118
			TRCN0000073119
			TRCN0000073120
			TRCN0000073121
			TRCN0000073122
9320	NM_004238	TRIP12	TRCN0000022374
			TRCN0000022375
			TRCN0000022376
			TRCN0000022377
			TRCN0000022378
26262	NM_130465	TSPAN17	TRCN0000034304
			TRCN0000034305
			TRCN0000034306
			TRCN0000034307
			TRCN0000034308
7337	NM_000462	UBE3A	TRCN0000003368
			TRCN0000003369
			TRCN0000003370
			TRCN0000003371
			TRCN0000003372
89910	NM_130466	UBE3B	TRCN0000004775
			TRCN0000004776
			TRCN0000004777
			TRCN0000004778
			TRCN0000004779
9690	NM_014671	UBE3C	TRCN0000003399
			TRCN0000003400
			TRCN0000003401
			TRCN0000003402

9690	NM_014671	UBE3C	TRCN0000010783
9354	NM_004788	UBE4A	TRCN0000007395<br>TRCN0000007396<br>TRCN0000007397<br>TRCN0000007398<br>TRCN0000007399
10277	NM_006048	UBE4B	TRCN0000007545<br>TRCN0000007546<br>TRCN0000007547<br>TRCN0000007548<br>TRCN0000007549
22888	NM_014948	UBOX5	TRCN0000004437<br>TRCN0000004438<br>TRCN0000004439<br>TRCN0000004440<br>TRCN0000004441
197131	NM_174916	UBR1	TRCN0000003423<br>TRCN0000003424<br>TRCN0000003425<br>TRCN0000003426<br>TRCN0000003427
23304	NM_015255	UBR2	TRCN0000003403<br>TRCN0000003404<br>TRCN0000003405<br>TRCN0000003406<br>TRCN0000003407
23352	NM_020765	UBR4	TRCN0000154749<br>TRCN0000155927<br>TRCN0000157658<br>TRCN0000157737

23352	NM_020765	UBR4	TRCN0000152115
			TRCN0000154429
			TRCN0000154886
			TRCN0000155202
			TRCN0000155617
115426	NM_152306	UHRF2	TRCN0000003479
			TRCN0000003480
			TRCN0000003481
			TRCN0000003482
			TRCN0000010793
7428	NM_000551	VHL	TRCN0000010459
			TRCN0000010460
			TRCN0000010461
			TRCN0000039623
			TRCN0000039624
			TRCN0000039625
			TRCN0000039626
			TRCN0000039627
151525	NM_152528	WDSUB1	TRCN0000073203
			TRCN0000073204
			TRCN0000073205
			TRCN0000073206
			TRCN0000073207
11059	NM_007013	WWP1	TRCN0000003395
			TRCN0000003396
			TRCN0000003397
			TRCN0000003398
			TRCN0000010782
11060	NM_007014	WWP2	TRCN0000001512
			TRCN0000001513

11060	NM_007014	WWP2	TRCN0000001514<br>TRCN0000001515<br>TRCN0000001516
10444	NM_006336	ZER1	TRCN0000139495<br>TRCN0000139522<br>TRCN0000140548<br>TRCN0000140793<br>TRCN0000143404<br>TRCN0000121635<br>TRCN0000122249
10444	NM_006336	ZER1	TRCN0000144077<br>TRCN0000144439<br>TRCN0000144625
130507	NM_172070	ZNF650	TRCN0000034099<br>TRCN0000034100<br>TRCN0000034101<br>TRCN0000034102<br>TRCN0000034103

Table S2. Mass spectrometry results for the series coimmunoprecipitated proteins associated with the Flag-NEDD4L and HA-Ubiquitin.

Uniprot accession	Protein name	kDa	Score	Unique peptides
Q4VCS5	Angiomotin (AMOT)	118.47	936	30
Q9BQA1	Methylosome protein 50 (WDR77)	37.44	344	8
**O14744**	**Protein arginine N-methyltransferase 5 (PRMT5)**	**73.32**	**335**	**14**
P46934	E3 ubiquitin-protein ligase NEDD4 (NEDD4)	150.28	322	7
P0CG47	Polyubiquitin-B (UBB)	25.80	229	6
Q8IY63	Angiomotin-like protein 1 (AMOTL1)	106.85	219	11
P15924	Desmoplakin (DSP)	334.02	163	6
P62258	14-3-3 protein epsilon (YWHAE)	29.32	94	3
Q969T9	WW domain-binding protein 2 (WBP2)	28.18	76	3
Q15018	BRISC complex subunit Abro1 (FAM175B)	47.1	62	2
Q9HCE7	E3 ubiquitin-protein ligase SMURF1 (SMURF1)	86.92	57	2
Q9NV92	NEDD4 family-interacting protein 2 (NDFIP2)	36.71	52	2
P07355	Annexin A2 (ANXA2)	38.81	43	3
Q8TER0	Sushi, nidogen and EGF-like domain-containing protein 1 (SNED1)	158.21	38	3
Q6P3W7	SCY1-like protein 2 (SCYL2)	104.33	37	1
Q06830	Peroxiredoxin-1 (PRDX1)	22.32	35	2
Q5VX52	Spermatogenesis-associated protein 1 (SPATA1)	50.50	27	1
Q02880	DNA topoisomerase 2-beta (TOP2B)	184.12	27	1
P20674	Cytochrome c oxidase subunit 5A, mitochondrial (COX5A)	16.92	27	1
O43150	Arf-GAP with SH3 domain, ANK repeat and PH domain-containing protein 2 (ASAP2)	112.84	26	1
Q9Y2J4	Angiomotin-like protein 2 (AMOTL2)	85.94	25	2
Q99943	1-acyl-sn-glycerol-3-phosphate acyltransferase alpha (AGPAT1)	32.04	25	1

Table S3. The list of primers.

shRNA recovered primers	
**Primer**	**Sequence**
shRNA-F	5’-GAGGGCCTATTTCCCATGAT-3’
shRNA-R	5’-GACGTGAAGAATGTGCGAGA-3’
**qPCR primers**	
**Primer**	**Sequence**
human NEDD4L-F	5’-GACATGGAGCATGGATGGGAA-3’
human NEDD4L-R	5’-GTTCGGCCTAAATTGTCCACT-3’
human AKT1-F	5’-TCTATGGCGCTGAGATTGTG-3’
human AKT1-R	5’-TCTTAATGTGCCCGTCCTTG-3’
human AKT2-F	5’-CGGTTTTATGGTGCAGAGATTG-3’
human AKT2-R	5’-AGTCAGTGATCTTGATGTGGC-3’
human AKT3-F	5’-TGTGGATTTACCTTATCCCCTCA-3’
human AKT3-R	5’-GTTTGGCTTTGGTCGTTCTGT-3’
**TaqMan Gene Expression Assay**	
**Gene name**	**Assay ID**
CTNNA1	Hs00944794_m1
CTNNB1	Hs00355049_m1
JUP	Hs00158408_m1
CDH1	Hs01023894_m1
VIM	Hs00185584_m1
SOX9	Hs01001343_g1
POU5F1	Hs04260367_gH
TAZ	Hs00794094_m1
SLUG	Hs00950344_m1
NANOG	Hs04260366_g1
ACTB	Hs01060665_g1

Table S4. The list of antibodies.

Antibody	RRID	Imm. Animal	Clone	Cat.#	Vendor	Applications
Anti-NEDD4L	AB_1904063	Rabbit	--	4013	CST, USA	WB (1:1000)
Anti-β-actin	AB_476743	Mouse	AC-74	A5316	Sigma, USA	WB (1:1000)
Anti-Flag	AB_259529	Mouse	M2	F3165	Sigma, USA	WB (1:1000)
Anti-HA	AB_2770404	Mouse	2S8Z1	AE008	Abclonal, China	WB (1:1000)
Anti-α-Catenin	AB_397592	Mouse	5	610193	BD, USA	WB (1:1000)
Anti-β-Catenin	AB_397554	Mouse	14	610153	BD, USA	WB (1:1000)
Anti-γ-Catenin	AB_397648	Mouse	15	610253	BD, USA	WB (1:1000)
Anti-E-Cadherin	AB_397580	Mouse	36	610181	BD, USA	WB (1:1000)
Anti-Vimentin	AB_393716	Mouse	RV202	550513	BD, USA	WB (1:1000)
Anti-p-mTOR	AB_10691552	Rabbit	D9C2	5536	CST, USA	WB (1:1000)
Anti-mTOR	AB_330978	Rabbit	--	2972	CST, USA	WB (1:1000)
Anti-tdTomoto	AB_2687917	Goat	--	orb182397	Biorbyt, UK	IF (1:300)
Anti-Ki67	AB_3072239	Rabbit	SR00-02	HA 721115	HUA BIO, China	IF (1:300)
Anti-p-4EBP1	AB_330947	Rabbit	--	9451	CST, USA	WB (1:1000)
Anti-4EBP1	AB_2097841	Rabbit	53H11	9644	CST, USA	WB (1:1000)
Anti-p-AKT	AB_2315049	Rabbit	D9E	4060	CST, USA	WB (1:3000)
Anti-AKT	AB_915783	Rabbit	C67E7	4691	CST, USA	WB (1:1000)
Anti-AKT	AB_1147620	Mouse	40D4	2920	CST, USA	WB (1:1000); IP (1:300)

Anti-Myc	AB_331783	Mouse	9B11	2276	CST, USA	WB (1:1000)
Anti-PRMT5	AB_2762092	Rabbit	--	A1520	Abclonal, China	WB (1:3000); IP (1:300)
Anti-Ubiquitin	AB_628423	Mouse	P4D1	sc-8017	Santa Cruz, USA	WB (1:200)
Anti-WDR77	AB_2772891	Mouse	AMC 0495	A9921	Abclonal, China	WB (1:1000)
Anti-SDMA&MMA	AB_3095615	Rabbit	--	PTM-617	PTM BIO, China	WB (1:500)
Anti-AKT1	AB_3069857	Rabbit	ST05-09	ET1609-47	HUA BIO, China	WB (1:3000); IP (1:300)
Anti-AKT2	AB_3071190	Rabbit	--	HA 500091	HUA BIO, China	WB (1:1000)
Anti-Mouse IgG-HRP	AB_631736	Goat	--	sc-2005	Santa Cruz, USA	WB (1:20000)
Anti-Rabbit IgG-HRP	AB_631746	Goat	--	sc-2004	Santa Cruz, USA	WB (1:20000)
Anti-Rabbit lgG HRP	--	Mouse	--	M21006	Abmart, China	WB (1:1000)
Alexa Fluor™ 488-conjugated Donkey anti-rabbit IgG (H+L)	AB_2576217	Donkey	--	A21206	Thermo Fisher Scientific, USA	IF (1:500)
Alexa Fluor™ 568-conjugated Streptavidin	AB_2315774	--	--	S11226	Thermo Fisher Scientific, USA	IF (1:500)
Biotinylated horse anti-goat IgG	AB_2336123	Horse	--	BA-9500	Vector Labs, USA	IF (1:300)

IP, immunoprecipitation assay; IF, immunofluorescence experiments; WB, western blotting.

Table S5. The list of sgRNAs.

sgRNA	Sequence
human PRMT5 #1	5’-ATGAACTCCCTCTTGAAACG-3’
human PRMT5 #2	5’-CCCTTCTCCGTCCCCGAGTT-3’
human AKT1 #1	5’-GGGAGTACATCAAGACCTGG-3’
human AKT1 #2	5’-ACCGCGTCCTGCAGAACTCC-3’
human AKT2	5’-CTCTTCAGCAGGAAGTACCG-3’
